# HAT1/HDAC2 mediated ACSL4 acetylation confers radiosensitivity by inducing ferroptosis in nasopharyngeal carcinoma

**DOI:** 10.1038/s41419-025-07477-4

**Published:** 2025-03-06

**Authors:** Peijun Zhou, Xingzhi Peng, Kun zhang, Jin Cheng, Min Tang, Lin Shen, Qin Zhou, Dan Li, Lifang Yang

**Affiliations:** 1 Department of Oncology, Key Laboratory of Carcinogenesis and Cancer Invasion of Ministry of Education, National Clinical Research Center for Geriatric Disorders, Xiangya Hospital, Central South University, Changsha, China; 2 Cancer Research Institute, School of Basic Medicine Science, Central South University, Changsha, China; 3 Department of Life Science, College of Biology, Hunan University, Changsha, China

**Keywords:** Head and neck cancer, Radiotherapy

## Abstract

Protein acetylation modification plays important roles in various aspects of tumor progression. Ferroptosis driven by lethal lipid peroxidation is closely related to tumor development. Targeting ferroptosis has become a promising strategy. However, the crosstalk between protein acetylation and ferroptosis remains unclear. In present study, we found that the acetylation of acyl-CoA synthase long-chain family member 4 (ACSL4) enhances its protein stability and a double-edged sword regulation in nasopharyngeal carcinoma (NPC). On the one hand, ACSL4 could promote the malignant progress of tumors; on the other hand, it enhanced radiosensitivity by endowing NPC cells with ferroptosis-sensitive properties in vitro and in vivo. Mechanistically, histone acetyltransferase 1 (HAT1) directly promotes the acetylation of ACSL4 at lysine 383, and deacetylase sirtuin 3 (SIRT3) mediates the deacetylation of ACSL4. Meanwhile, another deacetylase histone deacetylase 2 (HDAC2) enhances ACSL4 acetylation through inhibiting the transcription of SIRT3. Acetylation of ACSL4 inhibits F-box protein 10 (FBXO10)-mediated K48-linked ubiquitination, resulting in enhanced protein stability of ACSL4. This study reveals the novel regulatory mechanism of ferroptosis-related protein from the perspective of protein acetylation, and provides a novel method for the radiosensitivity of NPC.

## Introduction

Nasopharyngeal carcinoma (NPC) is a malignant tumor originating from nasopharyngeal mucosal epithelial cells. The incidence of NPC in southern China and Southeast Asia reaches up to 30/100,000 [1]. The main treatment of NPC is radiotherapy or concurrent chemoradiotherapy. In recent years, with the advancement of treatment technology, the local control rate of NPC has been significantly improved, but radioresistance always results in tumor recurrence and metastasis, making clinical treatment failure [2]. Therefore, it is meaningful to elucidate the molecular mechanisms of radioresistance and find effective methods to enhance radiosensitivity.

Ferroptosis is a novel regulatory cell death [3], which is principally characterized by increased intracellular redox iron content and accumulation of lipid peroxidation products [4]. Recent studies have found that ferroptosis is closely related to tumor development and radioresistance. In NPC, m6A demethylase FTO promotes radioresistance by inhibiting OTUB1-mediated ferroptosis [5]. The depletion of SOD2 can further enhance the radiosensitivity of NPC cells through ferroptosis induced by DHODH inhibition [6]. circADARB1 is significantly upregulated in NPC tissues and promotes radioresistance in NPC cells by inhibiting ferroptosis [7]. Therefore, an in-depth understanding of the regulatory mechanism and functions of ferroptosis will provide new and effective means to improve the radiotherapeutic effect of malignant tumors.

Protein post-translational modifications (PTMs) play an important role in various stages of tumor progression [8]. Acetylation modification is a dynamically reversible PTM that occurs in histones or non-histone proteins, and is collectively regulated by acetyltransferases and deacetylases [9]. Histone acetylation can affect transcription by altering chromatin structure or recruiting acetyl-lysine reader proteins [10]. Acetylation of non-histone proteins have been reported to participate in the malignant progression of tumors by regulating enzyme activity, protein stability, and changing protein subcellular localization as well as interactions with other proteins [11]. In recent years, studies have found that protein acetylation modification is involved in the regulation of ferroptosis. For example, EP300-mediated acetylation of HSPA5 at K353 suppresses GPX4 and promotes ferroptosis in pancreatic cancer cells [12]. GINS4 upregulates Snail expression, antagonizing p53 acetylation and thereby inhibiting ferroptosis in lung adenocarcinoma [13]. However, the interaction between protein acetylation and ferroptosis in NPC remains unclear.

Previously, we analyzed acetylation modified proteins in NPC cells used liquid chromatography-tandem mass spectrometry (LC-MS/MS) [14]. In this study, we further selected the ferroptosis-related protein ACSL4 which is highly expressed and acetylated in NPC. We found that ACSL4 could promote the malignant progression of NPC, and enhanced the tumor radiosensitivity through ferroptosis. Mechanistically, HAT1/SIRT3 dynamically regulated acetylation of ACSL4 at K383, and its acetylation could inhibit FBXO10-mediated K48-linked ubiquitination, resulting in enhanced protein stability of ACSL4.

## Materials and methods

### Plasmids and reagents

PCDH-CMV-Flag (#167463), pcDNA3.1-Flag (#20011), and pcDNA3.1-His (#52534) vectors were obtained from Addgene, pLVX-shRNA1 (632177) vector was purchased from Clontech (Mountain View, CA). HA-Ub-WT, HA-Ub-K48R (K48 mutation), HA-Ub-K63R (K63 mutation) plasmids were previously constructed by our laboratory [15]. About plasmid construction, pcDNA3.1-Flag vector was digested by ApaI (1604, Takara, Tokyo, Japan) and EcoRI (1611, Takara), and ACSL4 coding sequence was inserted into the digested vector by homologous recombination kit (C214, Vazyme, Nanjing, China) to obtain ACSL4 expression plasmid pcDNA-Flag-ASCL4. pcDNA3.1-His vector was double digested with ApaI (1604, Takara) and EcoRI (1611, Takara), the coding sequences of SIRT3, HAT1, and FBXO10 were respectively inserted into the digested vector by homologous recombination kit (C214, Vazyme) to obtain recombinant plasmids pcDNA-His-SIRT3, pcDNA-His-HAT1, and pcDNA-His-FBXO10. The PCDH-CMV-Flag vector was digested by BamHI (1605, Takara) and EcoRI (1611, Takara), and the ACSL4 coding sequence was inserted into the digestion vector by homologous recombination kit (C214, Vazyme) to obtain the PCDH-Flag-ACSL4 plasmid. The K383 acetylation (pcDNA-Flag-ASCL4-K383Q, PCDH-Flag-ASCL4-K383Q) and deacetylation (pcDNA-Flag-ASCL4-K383R, PCDH-Flag-ASCL4-K383R) mutant plasmids of pcDNA-Flag-ASCL4 and PCDH-Flag-ACSL4 plasmids were respectively constructed using point mutation kit (C215, Vazyme). Using pcDNA-His-SIRT3, the SIRT3 enzyme activity deletion plasmid pcDNA-His-SIRT3-H248Y was constructed [16]. Primers for homologous recombination were listed in Table S1. pLVX-shRNA1 plasmid double digested with BamHI (1605, Takara) and EcoRI (1611, Takara), and then negative control (NC), ACSL4, HAT1, HDAC1, HDAC2, HDAC3, SIRT3 interference sequence were respectively inserted into the digested vector to obtain NC plasmid, knockdown ACSL4 plasmids (shACSL4#1, shACSL4#2), HAT1 plasmids (shHAT1#1, shHAT1#2), HDAC1 plasmid (shHDAC1), HDAC2 plasmid (shHDAC2), HDAC3 plasmid (shHDAC3) and SIRT3 plasmid (shSIRT3). The interference sequences were listed in Table S2.

LBH589 (S1030), Nicotinamide (NAM, S1899), Santacruzamate A (STA, S7595), RSL3 (S8155), Ferrostatin-1 (Fer-1, S7243), and 3-TYP (S8628) were purchased from Selleck Chemicals (Houston, TX). LMK-235 (HY-18998), MS-275 (HY-12163), MG-132 (HY-13259), and CHX (HY-12320) were obtained from MedChemExpress (Monmouth Junction, NJ).

### Cell culture and transfection

All NPC cell lines (HK1, SUNE1, 5-8 F, 6-10B, HONE1, C666-1, CNE1), normal nasopharyngeal epithelial cell NP69, and 293 T cells were obtained from the Cell Center of Central South University. HK1, SUNE1, 5-8 F, 6-10B, HONE1, C666-1, and CNE1 cells were cultured in RPMI 1640 (Gibco BRL, Grand Island, NY) supplemented with 10% fetal bovine serum (FBS, Hyclone, South Logan, UT). 293 T cells were cultured in DMEM (Gibco BRL) supplemented with 10% FBS (Hyclone). Normal nasopharyngeal epithelial cells NP69 were cultured in Defined K-SFM (Gibco BRL) medium at 37 °C in an incubator containing 5% CO_2_.

Plasmid transfection was performed using Neofect (TF201201, Neofect Biotech, Beijing, China) according to the manufacturer’s instructions. For the construction of stable cell lines, the constructed NC, shACSL4#1, shACSL4#2, shHAT1#1, shHAT1#2, shSIRT3, PCDH-CMV-Flag, PCDH-Flag-ACSL4, PCDH-Flag-ACSL4-K383R, PCDH-Flag-ACSL4-K383Q plasmids were respectively transfected into 293 T cells with the lentiviral expression system (PT5135-1, Takara), and the supernatant of 293 T cells was collected after 48 h. Subsequently, the supernatant was co-cultured with HK1, SUNE1 or HONE1 cells for 48 h, and the cells were screened with Puromycin (ST551, beyotime, Shanghai, China) to obtain HK1-NC, HK1-shACSL4#1, HK1-shACSL4#2, SUNE1-NC, SUNE1-shACSL4#1, SUNE1-shACSL4#2, SUNE1-shHAT1#1, SUNE1-shHAT1#2, SUNE1-shACSL4-vec, SUNE1-shACSL4-WT, SUNE1-shACSL4-K383R, SUNE1-shACSL4-K383Q, HONE1-NC, HONE1-shACSL4#1, HONE1-shACSL4#2, HONE1-shHAT1#1, HONE1-shHAT1#2, HONE1-shSIRT3, HONE1-vec, HONE1-ACSL4-WT, HONE1-ACSL4-K383R, and HONE1-ACSL4-K383Q cells, respectively.

### Quantitative PCR (qPCR)

According to the manufacturer’s instructions, total RNA was extracted using a total RNA extraction kit (15596026, Thermo Fisher Scientific, Waltham, MA), and the cDNA was further obtained using a reverse transcription kit (K1621, Thermo Fisher Scientific). qPCR analysis was performed using SYBR Green (4309155, Life Technologies Corporation, Gaithersburg, MD) on an ABI 7500 (Foster city, CA) instrument. The primer sequences were shown in Table S3.

### Western blot and immunoprecipitation (IP)

Cells were lysed in IP buffer (87787, Thermo Fisher Scientific) containing inhibitor cocktail (4693116001, Roche, Basel, Switzerland) and phosphatase inhibitor (4906845001, Roche). Then centrifuged at 13,000 rpm for 15 min at 4 °C. According to the manufacturer’s instructions, BCA reagent (AR0197, Boster Biological Technology, Wuhan, China) was used to determine the protein concentration and boiled with 5× loading buffer. The sample was loaded onto SDS-PAGE gel and transferred to a polyvinylidene fluoride membrane (Merck Millipore, Billerica, MA). After blocking, the blots were incubated with primary antibodies at 4 °C overnight and then incubated with secondary antibodies for 1 h at room temperature. The bands were observed by enhanced chemiluminescence detection kit (36208-A, Yeasen, Shanghai, China). For IP, cell lysates were incubated overnight with magnetic beads (10004D, Thermo Fisher Scientific) and antibodies at 4 °C. Then the magnetic beads were washed three times with lysis buffer and used for subsequent western blot. About antibodies, ACSL4 (22401-A-AP), SIRT3 (10099-1-AP), HAT1 (11432-1-AP), His (66005-1-Ig), Flag (80010-1-RR), Ubiquitin (10201-2-AP) were purchased from Proteintech (Chicago, IL). FBXO10 (A14871) and β-actin (AC026) were purchased from ABclonal (Wuhan, China). HA (#2367), HDAC2 (#5113S) anti-rabbit IgG-HRP (14708) and anti-mouse IgG-HRP (14709) antibodies were obtained from Cell Signaling Technology (Danvers, MA). HDAC1 (WL01297) and HDAC3 (WL02946) were purchased from Wanleibio (Shenyang, China). Acetyl lysine (ab21623) was purchased from Abcam (Cambridge, MA).

### Ubiquitination assay

Indicated plasmids were co-transfected into 293 T cells or NPC cells, then treated with MG-132 (20 μM) for 10 h. Cells were lysed in IP buffer (87787, Thermo Fisher Scientific) containing inhibitor cocktail (4693116001, Roche) and phosphatase inhibitor (4906845001, Roche). After lysates boiled for 10 min, dilution buffer (10 mM Tris·HCl, pH 8.0, 150 mM NaCl, 2 mM EDTA, 1% Triton) was added and the samples were centrifuged to obtain the cytosolic protein fraction followed by incubating with Flag (80010-1-RR) or ACSL4 (22401-A-AP) antibodies overnight at 4 °C, then added 30 μL of magnetic beads (10004D, Thermo Fisher Scientific). The beads-antibody-protein complexes were washed with cold lysis buffer and boiled for 10 min using 5 × SDS-PAGE loading buffer, and analyzed using western blot with ubiquitin or HA antibodies.

### Immunoprecipitation (IP) and mass spectrometry (MS) analysis

SUNE1 cells were lysed in IP buffer (87787, Thermo Fisher Scientific) containing inhibitor cocktail (4693116001, Roche) and phosphatase inhibitor (4906845001, Roche), then centrifuged at 13,000 rpm at 4 °C for 15 min. Protein concentration was determined by BCA reagent (AR0197, Boster Biological Technology). Subsequently, the cell lysates were incubated with magnetic beads and ACSL4 antibodies at 4 °C overnight. The magnetic beads were washed three times and sent to PTM bio (Hangzhou, China) for mass spectrometry identification using a mass spectrometer (TMQ Exactive Plus, Thermo Fisher Scientific).

### Bioinformatics analysis

The iProX database (https://www.iprox.cn/) is a comprehensive proteome resource center in China, where the IPX0001265000 contains proteome data from tumor samples of 30 patients with NPC and nasopharyngeal tissue samples of 22 tumor-free individuals [17]. GSE12452 contains transcriptomic data from 31 cases of NPC and 10 cases of normal nasopharyngeal tissues [18]. Uniprot database (https://www.uniprot.org/) was used to analyze the conservation of potential acetylation sites of ACSL4. KOBAS database (http://kobas.cbi.pku.edu.cn/) was used for KEGG enrichment analysis of differential genes. About protein molecular docking analysis, SWISS-MODEL database (https://swissmodel.expasy.org/) is used to obtain the PDB files of ACSL4, ACSL4-K383R, ACSL4-K383Q, SIRT3, HAT1, FBXO10. The Vakser Lab-GRAMM Web tool (https://gramm.compbio.ku.edu/) was used to analyze the molecular docking of SIRT3, HAT1, FBXO10, and ACSL4. PyMOL software visualizes these docking structures in cartoon, surface, or rod form.

### Cell viability assay

Cell viability was measured by CCK8 kit (C0005, Targetmol, Boston, MA) according to the manufacturer’s instructions. The cells in were cultured in 96-well plates for a specified time, and then the determination solution was added and incubated for 2 h in the dark. Microplate Reader (BioTek ELx800, Winooski, VT) was used for measurement at 450 nm.

### Colony formation assay

The same number of cells in the control group and treated group were inoculated into a 6-well plate, and further cultured for 15 days to form colonies after being attached to the plate. When analyzing radiosensitivity, cells were irradiated with X-rays (0, 1, 2, 4, or 6 Gy) after cell attachment, and then cultured for 15 days to form colonies. Subsequently, the colonies in the culture dish were stained with crystal violet solution (V5265, Sigma-Aldrich, St. Louis, MO), and the number of viable colonies (defined as colonies with >50 cells) was calculated.

### X-ray ionizing radiation (IR)

The cells and BALB/C nude mice IR experiments were carried out on a PXI X-RAD 225 system (Precision X-ray Inc., North Branford, CT) at indicated dosages.

### Scratch assay

After the cells were adherent and the density reached 100%, the cells were scratched with a 200 μL pipette tip, and the isolated cells were removed with PBS. At the appropriate time, photographs were taken using a phase contrast microscope (AMEX-1200, AMG, Bothell, WA) (×100), and Image J was used to calculate the area.

### Transwell assay

Transwell assays were performed using 24-well plates and transwell inserts (BD Biosciences, SanDiego, CA) were coated with Matrigel (356234, Corning, NY). Firstly, 1 × 10^5^ cells were added to the upper chamber of 0.2 mL serum-free medium, and 0.8 mL medium containing 10% FBS was added to the lower chamber. After 24 h of incubation, invasive cells attached to the bottom surface of the filter were stained with crystal violet solution (V5265, Sigma-Aldrich), and observed and photographed under a microscope (Leica DMI 3000B, Germany).

### Analysis of lipid ROS

Cells were cultured in PBS containing 10 μM BODIPY TM 581/591 C11(#D3861, Thermo Fisher Scientific) at 37 °C cell for 30 min. Subsequently, cells were collected by trypsin (WLA094A, Wanleibio) digestion, and resuspended in 500 μL fresh PBS. Lipid ROS levels were analyzed by a flow cytometry (Fortessa, BD Biosciences, San Jose, CA).

### Transmission electron microscope (TEM)

NPC cells treated with DMSO or RSL3 were collected and fixed with 2.5% glutaraldehyde (AWI0097, abiowell, Changsha, China), and then sent to Wellbio (Changsha, China). Transmission electron microscopy (JEM1400, JEOL, Tokyo, Japan) was used to observe and collect pictures.

### Immunofluorescence (IF)

For the exogenous IF experiment, cells were transfected with pcDNA-His-HAT1, pcDNA-His-SIRT3, pcDNA-His-FBXO10 or pcDNA-Flag-ACSL4 plasmids. Flag (80010-1-RR, Proteintech) and His (66005-1-Ig, Proteintech) antibodies were incubated at 37 °C for 1 h. For the endogenous IF experiment, SIRT3 (10099-1-AP, Proteintech), HAT1 (11432-1-AP, Proteintech), or FBXO10 (A14871, ABclonal) were co-incubated with ACSL4 (sc-271800, Santa Cruz, CA) antibody at 37 °C for 1 h. Then Anti-Rabbit-CF TM 488 A (SAB4600234, Sigma-Aldrich) and Anti-Mouse-Alexa Fluor TM 594 (A-11005, Thermo Fisher Scientific) were incubated at 37 °C for 1 h. Confocal microscopy (LSM 510 META, Carl Zeiss, Germany) was used to observe and collect images.

### Animal experiments

All experimental animals were 5-week-old female athymic nude mice (BALB/C). To explore the effect of HAT1 on the growth of NPC, 5 × 10^6^ SUNE1-NC cells or SUNE1-shHAT1 cells were subcutaneously injected into nude mice to establish the xenograft model (*n* = 6). For HDAC2, after injection with 5 × 10^6^ SUNE1 cells, 12 nude mice were randomly divided into two groups. The control group was intraperitoneally injected with DMSO (100 μL), and the treatment group was intraperitoneally injected with 100 μL HDAC2 inhibitor STA (50 mg/kg) dissolved in DMSO, every other day for two weeks. To investigate the effect of ACSL4 on the radiosensitivity, 24 nude mice were randomly divided into two groups. One group was subcutaneously injected with 5 × 10^6^ SUNE1-NC cells, while the other group was injected with 5 × 10^6^ SUNE1-shACSL4 cells. Subsequently, 6 mice from each group were randomly selected for IR treatment with 2 Gy, once every 4 days, for two weeks. To explore the role of ACSL4-mediated ferroptosis in regulating NPC radiosensitivity, 48 nude mice were subcutaneously injected with either 5 × 10^6^ SUNE1-NC cells or 5 × 10^6^ SUNE1-shACSL4 cells. Following randomization into subgroups (*n* = 6), the control group was intraperitoneally injected with 100 μL DMSO, while the treatment group was intraperitoneally injected with 100 μL RSL3 (100 mg/kg) dissolved in DMSO. The RSL3 injections were administered on three consecutive days, followed by a one-day interval, for two weeks. The IR treatment was the same as above. To investigate the effect of ACSL4 deacetylation on ferroptosis and radiosensitivity, 48 nude mice were subcutaneously injected with either 5 × 10^6^ SUNE1-shACSL4-WT or 5 × 10^6^ SUNE1-shACSL4-K383R cells. After randomization into subgroups (*n* = 6), the treatment of RSL3 or IR was similar as above.

All experimental animals were examined and measured once every other day. The tumor volume was calculated as volume (mm^3^) = d^2^ × D/2, where d and D were the shortest and longest diameters, respectively. The animals were sacrificed at the indicated time point, and the tumor tissues were collected and weighed, and fixed with 10% formalin for immunohistochemical analysis.

### Immunohistochemistry (IHC)

Collected tissue sections of animal tumor and paraffin-embedded 33 nasopharyngeal normal tissues and 40 NPC tissue with clinical details of patients in the Xiangya Hospital, Central South University (2021–2022) (Table S4). According to the manufacturer’s instructions, IHC was performed using the universal two-step detection kit (PV-9000, ZSGB-BIO) and the DAB kit (ZLI-9017, ZSGB-BIO, Beijing, China). The antibodies of 4-HNE (MAB3249, R&D Systems, Minneapolis, MN) and Ki67 (27309-1-AP, Proteintech, Chicago, IL, USA) were used. All immunostained sections were then counterstained with hematoxylin (E607317, Sangon bio, Shanghai, China). The expression of each protein in IHC was semi-quantitatively evaluated using the previously described method [19].

### Statistical analysis

Statistical analysis was performed using GraphPad Prism 9 (SanDiego, California). The data are expressed as mean ± SD. Differences between groups were assessed using a two-tailed student *t*-test, and a *p*-value < 0.05 was considered statistically significant.

## Results

### HDAC2/SIRT3 mediates acetylation and high expression of ACSL4 in NPC

**Fig. 1:**
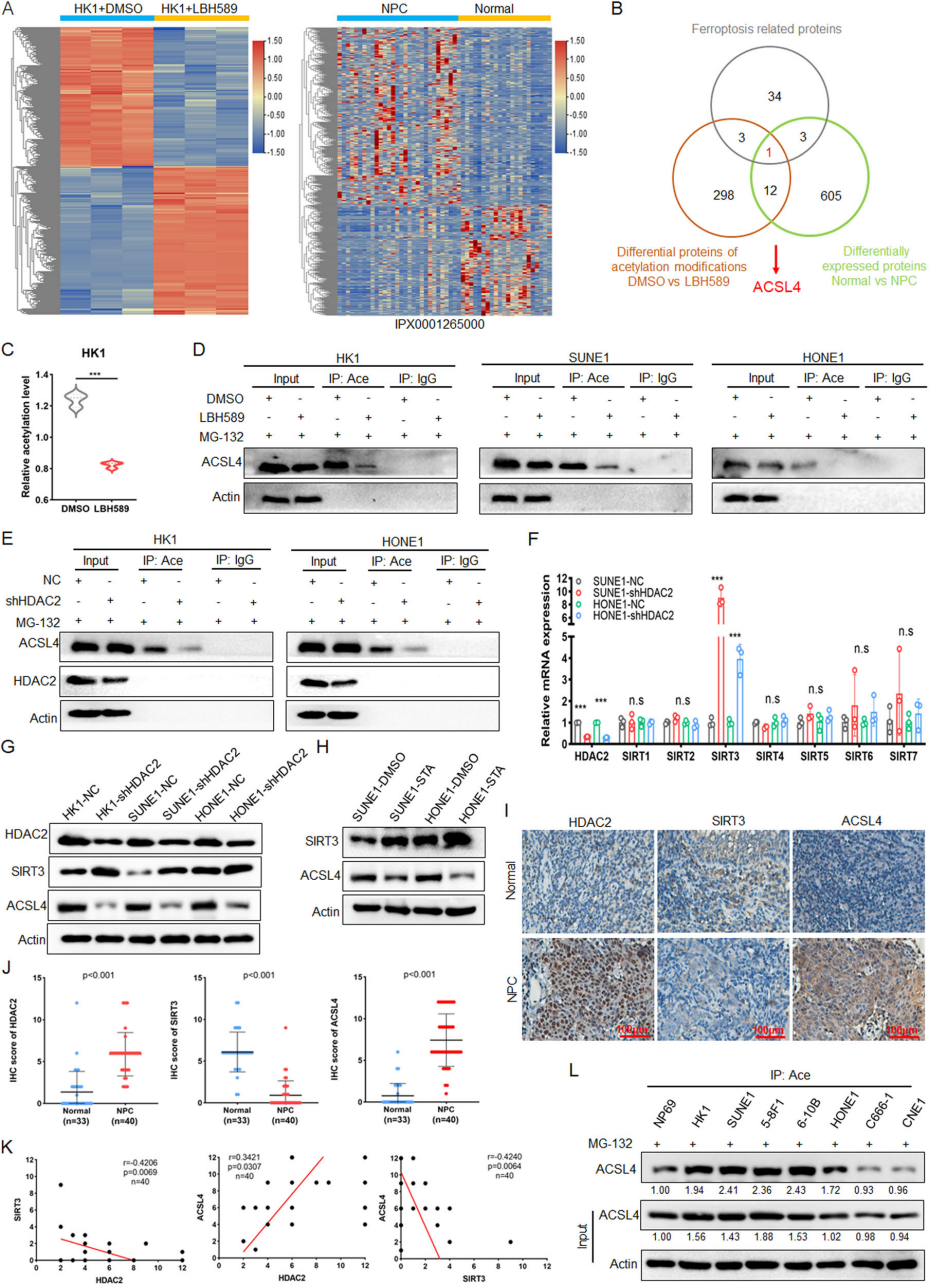
HDAC2/SIRT3 mediates the acetylation and high expression of ACSL4 in NPC. **A** Heat map showed acetylated differential proteins in HK1 cells after LBH589 treatment (|Fold Change| >1.5, *p* < 0.05) (left) and differential proteins in normal nasopharyngeal tissues and NPC samples proteomics data IPX0001265000 (|Fold Change| > 2) (right). **B** Venn diagram was used to analyze the intersection of acetylated differential proteins, NPC proteomics differential proteins and KEGG ferroptosis pathway molecules. **C** The statistical diagram of ACSL4 acetylation by acetylation proteomics. **D** NPC cells were treated with LBH589 (100 nM) for 24 h, and then treated with MG-132 (20 μM) for 24 h. The acetylation of ACSL4 was detected by IP assay. After knocking down HDAC2 in NPC cells, **E** the acetylation of ACSL4 was detected by IP assay, **F** the mRNA levels of HDAC2 and SIRT1-7 were detected by qPCR, **G** the protein expression of HDAC2, SIRT3 and ACSL4 were detected by western blot. **H** NPC cells were treated with STA (20 μM) for 24 h, and the protein expression of SIRT3 and ACSL4 were detected by western blot. **I** The representative IHC images of HDAC2, SIRT3, and ACSL4 in 40 cases of NPC and 33 cases of normal clinical nasopharyngeal samples. **J** Statistical analysis of protein expression by IHC scores. **K** Correlation analysis for the expression of HDAC2, SIRT3, and ACSL4 by IHC scores. **L** After NPC cells and normal nasopharyngeal epithelial cells were treated with MG-132 (20 μM) for 24 h, IP experiments were used to detect acetylation of ACSL4. The error line is expressed as mean ± SD. No significant (ns), *** *p* < 0.001.

To identify the acetylated non-histone proteins in NPC cells, we used LC-MS/MS to analyze the acetylated differential proteins in NPC cells HK1 after treated with pan-HDAC inhibitor LBH589 [14]. The results displayed that acetylation was upregulated at 240 sites of 140 proteins, and downregulated at 228 sites of 186 proteins (|Fold Change| >1.5, *p* < 0.05) (Fig. 1A left). Then the online IProX proteomics database IPX0001265000 was used to analyze the differential proteins between nasopharyngeal normal tissues and NPC samples. There were 383 proteins upregulated and 238 proteins downregulated in NPC (|Fold Change| > 2, *p* < 0.05) (Fig. 1A right). KEGG analysis of acetylated differential proteins (Fig. S1A) and NPC differentially expressed proteins (Fig. S1B) revealed that differential molecules could be enriched in the ferroptosis pathway. Further analysis of the Venn diagram revealed that ACSL4 was the only ferroptosis-related protein that had both acetylation modification and differential expression (Fig. 1B). The results of acetylation proteomics showed that LBH589 could significantly inhibit the acetylation of ACSL4 (Fig. 1C). In NPC cell lines HK1, SUNE1, and HONE1, IP experiments using pan-acetylated lysine antibody showed that LBH589 not only inhibited ACSL4 acetylation but also inhibited its expression (Fig. S2A). To avoid the influence of protein expression changes on ACSL4 acetylation, we simultaneously treated cells with the proteasome inhibitor MG-132 in addition to LBH589. The results similarly demonstrated that LBH589 could inhibit the acetylation level of ACSL4 (Fig. 1D). The expression of ACSL4 mRNA after LBH589 treatment was detected in NPC cells, and it was found that the mRNA level of ACSL4 did not change significantly (Fig. S2B), suggesting that LBH589 affects ACSL4 protein expression through PTM.

Next, we explored which deacetylase mediates the regulation of LBH589 on ACSL4 protein expression. After NPC cells were treated with class I HDAC inhibitor MS-275 and class II HDAC inhibitor LMK-235, the results showed that MS-275 could inhibit the expression of ACSL4 (Fig. S2C). Further, knocking down MS-275 targets HDAC1, HDAC2, and HDAC3 respectively, it was found that only knocking down HDAC2 could significantly inhibit the protein expression of ACSL4 (Fig. S2D), and IP experiments showed that knockdown HDAC2 could inhibit ACSL4 acetylation (Fig. S2E), Meanwhile, IP experiments performed with MG-132 treatment under the same conditions also reached the same conclusion (Fig. 1E). these results indicated that LBH589 inhibits acetylation and expression of ACSL4 by inhibiting HDAC2. As a deacetylase, why HDAC2 promoted the acetylation of ACSL4? Studies have shown that HDAC2 can bind and cause histone deacetylation in the promoter regions of class III HDACs SIRT3 and SIRT7, reducing their transcriptional activity, thus downregulating the expression of SIRT3 and SIRT7 [20, 21]. The results of western blot showed that the treatment of class III HDAC inhibitor NAM could promote the protein expression of ACSL4 in NPC cells (Fig. S3A). These suggests that HDAC2 may promote the acetylation and expression of ACSL4 by inhibiting SIRTs. Therefore, qPCR results showed that among the seven SIRT family members, knockdown of HDAC2 was only able to upregulate the mRNA level of SIRT3 in NPC cells (Fig. 1F). Additionally, qPCR experiments were conducted after treating SUNE1 and HONE1 cells with LBH589, and the data showed that LBH589 significantly upregulated the mRNA expression of SIRT3 (Fig. S3B). Subsequently, it was also verified that knockdown HDAC2 could upregulate the protein expression of SIRT3 and reduce the protein expression of ACSL4 (Fig. 1G). Further, STA, a specific inhibitor of HDAC2, was found to upregulate SIRT3, and reduce the protein expression of ACSL4 (Fig. 1H). The analysis of GSE12452 dataset found that HDAC2 was highly expressed in NPC, while SIRT3 was lowly expressed, and HDAC2 was negative correlated with SIRT3 (Fig. S3C–E). Similarly, the proteomic data of NPC showed that HDAC2 and ACSL4 were highly expressed in NPC, while SIRT3 was lowly expressed (Fig. S3F–H). Consistent with this, the IHC analysis using clinical NPC and nasopharyngeal normal tissue samples revealed that HDAC2 and ACSL4 were upregulated in NPC, and SIRT3 was downregulated (Fig. 1I, J). The results of correlation analysis showed that the expression of HDAC2 was positively correlated with ACSL4, and the expression of SIRT3 was negatively correlated with HDAC2 and ACSL4 (Fig. 1K). These data suggested that HDAC2 may promote the expression of ACSL4 by inhibiting SIRT3. Additionally, the IP assay was performed in NPC cells and normal nasopharyngeal epithelial cell NP69. It was found that ACSL4 was highly acetylated and expressed in most NPC cells (Fig. S3I). Meanwhile, IP experiments performed with MG-132 treatment under the same conditions also reached the same conclusion (Fig. 1L). While the results of qPCR (Fig. S3J) and bioinformatics analysis (Fig. S3K) showed that the mRNA level of ACSL4 was not significantly upregulated in NPC. It is further explained that the high protein expression of ACSL4 is mainly caused by PTM. The above results indicated that the HDAC2/SIRT3 axis mediates the acetylation and high expression of ACSL4 in NPC.

### ACSL4 promotes the malignant progression of NPC

**Fig. 2:**
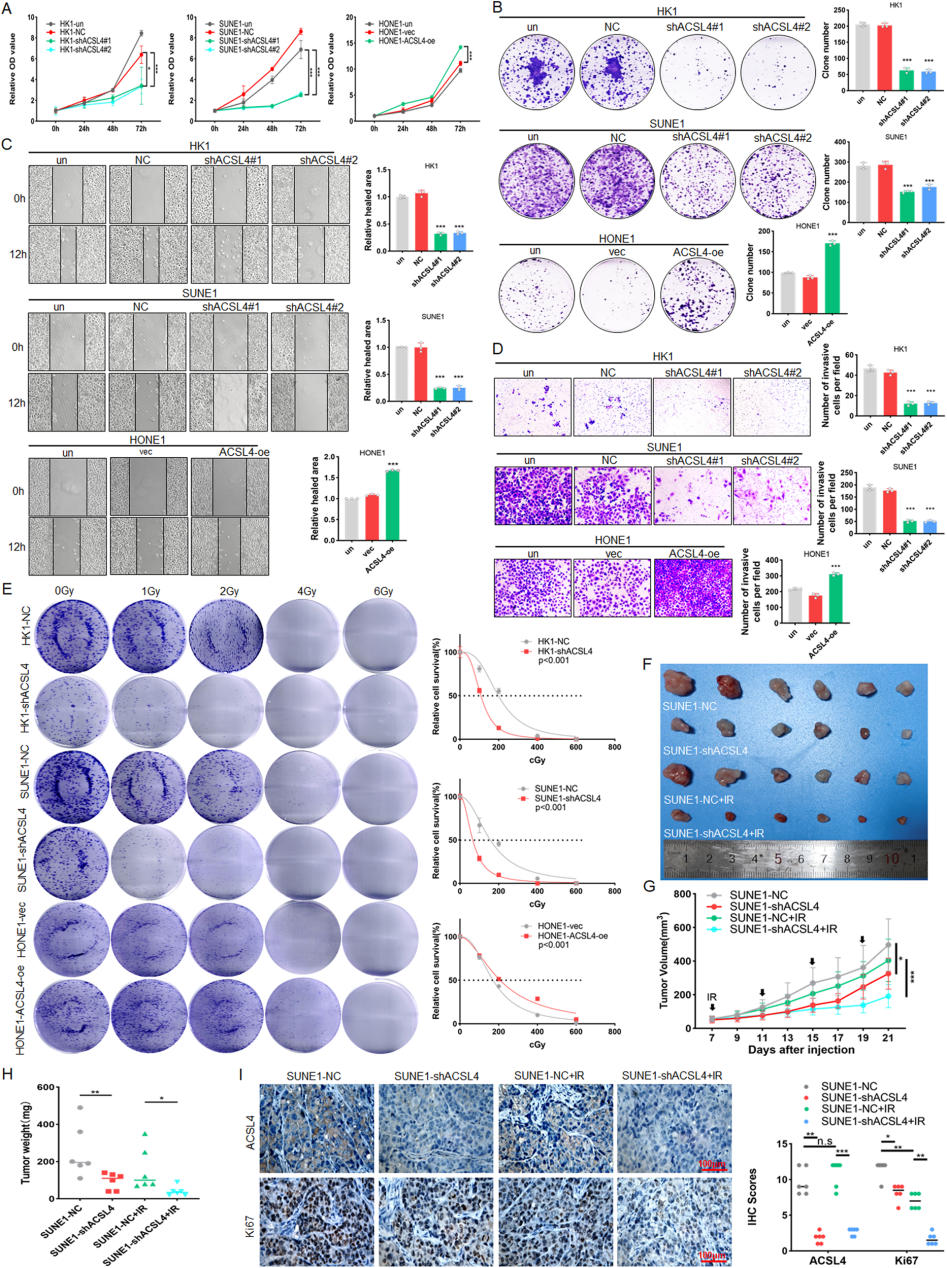
ACSL4 promotes the malignant progression of NPC. HK1, HK1-NC, HK1-shACSL4#1, HK1-shACSL4#2, SUNE1, SUNE1-NC, SUNE1-shACSL4#1, SUNE1-shACSL4#2 and HONE1, HONE1-vec, HONE1-ACSL4-oe cells were used. **A** CCK8 and **B** colony formation assay was used to detect the cell proliferation. **C** Scratch assay and **D** Transwell assay were used to detect the cell migration and invasion. **E** The radioresistance of NPC cells (2 × 10^3^ cells/well) was detected by colony formation assay under different doses (0, 1, 2, 4, 6 Gy) of IR. The 2 × 10^6^ SUNE1-shACSL4 cells or SUNE1-NC cells were injected subcutaneously into nude mice and randomly divided into non-IR group and IR group (*n* = 6). **F** Tumor size, **G** tumor volume and **H** tumor weight was measured. **I** IHC was used to detect the expression of ACSL4 and Ki67 in tumor tissues. Untreated group (un), negative control (NC), blank plasmid (vec). The error line is expressed as mean ± SD. *p < 0.05, **p < 0.01, ***p < 0.001.

To elucidate the role of ACSL4 in the malignant progression of NPC, HK1, SUNE1, HONE1 and respectively knockdown or overexpress ACSL4 cells (Fig. S4A) were used. The results of CCK8 and colony formation assays showed that knockdown of ACSL4 in HK1 and SUNE1 cells could inhibit cell proliferation, while overexpression of ACSL4 in HONE1 cells could promote cell proliferation (Fig. 2A, B). The results of scratch and transwell assays indicated that ACSL4 could promote cell migration and invasion of NPC cells (Fig. 2C, D). And the results of colony formation assay displayed that knockdown of ACSL4 could inhibit the radioresistance of NPC cells, while overexpression of ACSL4 had an opposite function (Fig. 2E). Meanwhile, to exclude the possible off-target effects of the shRNA. We conducted rescue experiments by overexpressing ACSL4 in ACSL4-knockdown HONE1 cells (Fig. S4B). And the results of CCK8 assays, transwell assays and radiation treatment-based colony formation assays indicated that ACSL4 indeed promotes the proliferation, invasion and radioresistance of NPC cells (Fig. S4C–E). Subsequently, SUNE1-NC and SUNE1-shACSL4 cells were used to establish the xenograft model and combined with IR treatment. The results revealed that knockdown of ACSL4 could inhibit the growth of NPC and promote the radiosensitivity of tumors (Fig. 2F–H). IHC analysis showed that knockdown of ACSL4 could inhibit the positive rate of Ki67, and combined with IR treatment could further reduce Ki67 stain (Fig. 2I). These results revealed that ACSL4 could promote proliferation, invasion, and radioresistance of NPC.

### ACSL4 enhances radiosensitivity by inducing ferroptosis

**Fig. 3:**
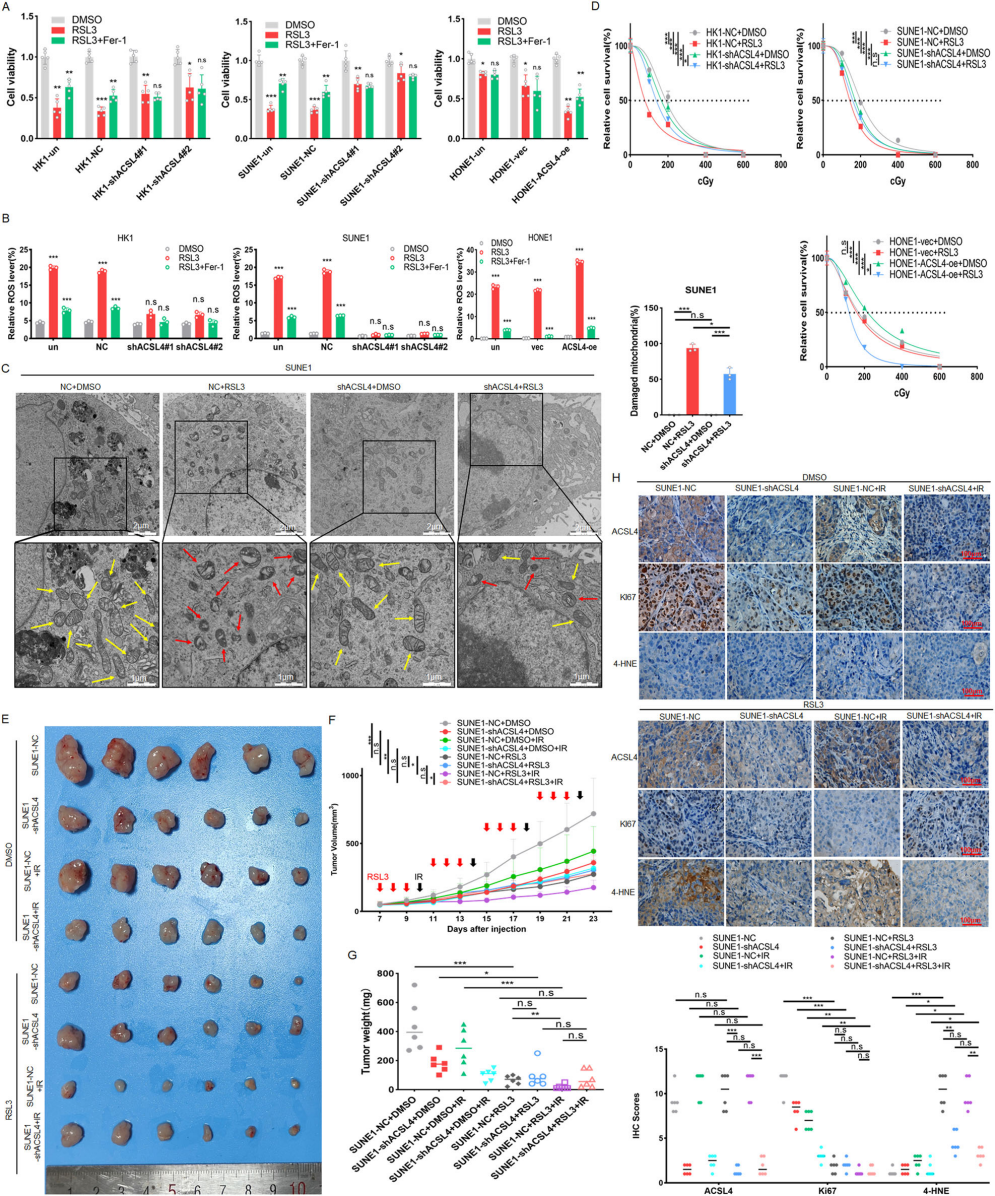
ACSL4 enhances radiosensitivity by inducing ferroptosis. The stable cell lines were treated with RSL3 (5 μM) or with both RSL3 and Fer-1(2 μM) for 24 h, **A** CCK8 assay was used to detect the cell viability. **B** The statistical diagram of lip-ROS levels detected by flow cytometry. **C** The representative images of mitochondrial morphology were showed by TEM (red arrows indicated mitochondria with shrinkage and cristae rupture, and yellow arrows indicated normal mitochondria). **D** The radioresistance of NPC cells (3 × 10^3^ cells/well) was detected by colony formation assay under different doses (0, 1, 2, 4, 6 Gy) of and IR treatment with RSL3 (5 μM). 2 × 10^6^ SUNE1-shACSL4 or SUNE1-NC cells were injected subcutaneously into nude mice and randomly divided into non-IR group and IR group, and then divided into DMSO treatment group and RSL3 treatment group (*n* = 6). **E** Tumor size, **F** tumor volume and **G** tumor weight was measured. **H** IHC was used to detect the expression of ACSL4, 4-HNE and Ki67 in tumor tissues. The error line is expressed as mean ± SD. Untreated group (un), negative control (NC), blank plasmid (vec), no significant (ns), *p < 0.05, **p < 0.01, ***p < 0.001.

Studies have shown that ACSL4 can promote cell ferroptosis by catalyzing fatty acid oxidation to produce acyl-CoA and increase of PUFA-containing lipids and the accumulation of membrane lipid peroxides [22]. After treated with the ferroptosis inducer RSL3, CCK8 results showed that NPC cells with knockdown of ACSL4 were resistant to RSL3. On the contrary, cells with overexpression of ACSL4 resulted in being more sensitive to RSL3 (Fig. S5A), and this effect was inhibited after treated with the ferroptosis inhibitor Fer-1 (Fig. 3A). Similarly, knockdown of ACSL4 reduced the increase of lip-ROS content induced by RSL3, while overexpression of ACSL4 promoted the induction of RSL3, and further Fer-1 treatment could inhibit this effect (Figs. 3B and S5B). Then we analyzed the ultrastructural changes of mitochondria by TEM. There were substantial typical mitochondria with abundant cristae in SUNE1 cells, after RSL3 treatment, plenty of mitochondria shrank and the number of cristae decreased significantly, while knockdown of ACSL4 could inhibit this effect (Fig. 3C), indicating that high expression of ACSL4 could promote ferroptosis of NPC cells. Studies have revealed that ferroptosis is involved in the radiotherapy of tumors, and inhibition of ferroptosis significantly impairs the efficacy of radiotherapy [23]. We then explored the effect of ACSL4-mediated ferroptosis on radiosensitivity of NPC in vivo and in vitro. Knockdown of ACSL4 leaded to radioresistance by inhibiting ferroptosis, while overexpression of ACSL4 enhanced radiosensitivity by promoting ferroptosis in tumor cells (Fig. 3D–G). IHC analysis showed that in the ACSL4 high expression group, RSL3 combined with IR treatment could significantly reduce Ki67 stain. After knocking down ACSL4, RSL3-induced ferroptosis marker 4-HNE was reduced (Fig. 3H), indicating that ACSL4-mediated ferroptosis can promote the radiosensitivity of NPC.

### HAT1 and SIRT3 directly mediate the acetylation modification of ACSL4

**Fig. 4:**
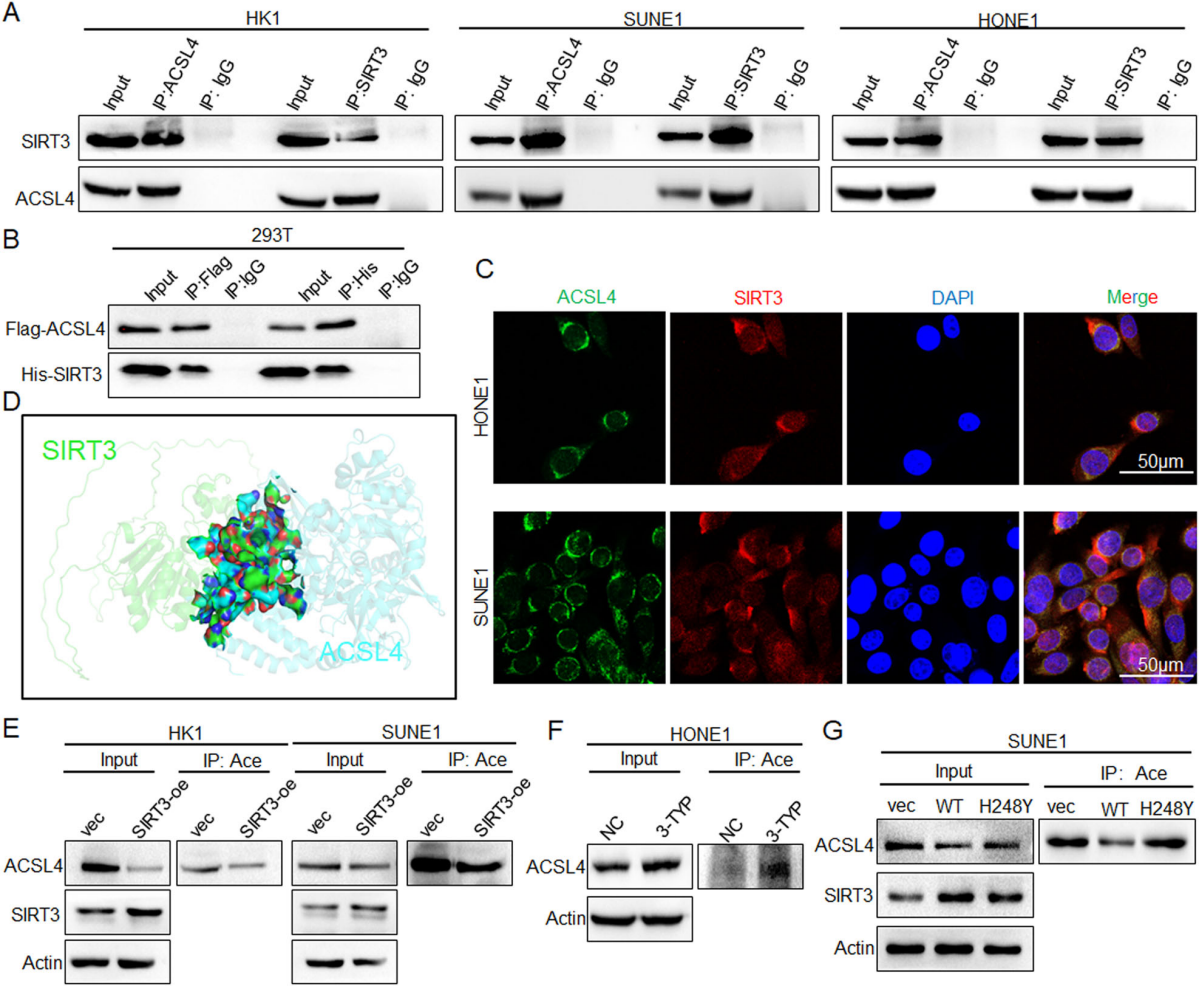
SIRT3 directly mediates the deacetylation of ACSL4. **A** IP assay was used to detect the interaction between ACSL4 and SIRT3 in NPC cells. **B** His-SIRT3 and Flag-ACSL4 plasmids were transfected into 293 T cells, and the interaction between ACSL4 and SIRT3 was detected by IP assay. **C** The subcellular localization of ACSL4 and SIRT3 was detected by IF assay. **D** Molecular docking analysis of the interaction between ACSL4 and SIRT3. **E** After SIRT3 was overexpressed in NPC cells, the acetylation and protein expression of ACSL4 were detected by IP assay. **F** HONE1 cells were treated with 3-TYP (50 μM) for 24 h, the acetylation of ACSL4 were detected by IP assay. **G** SIRT3-WT or SIRT3-H248Y plasmids were transfected into SUNE1 cells, and the acetylation and protein expression of ACSL4 were detected by IP assay. Blank plasmid (vec), negative control (NC).

To further clarify whether SIRT3 directly mediates the deacetylation of ACSL4, IP experiments revealed that ACSL4 had an endogenous interaction with SIRT3 in NPC cells (Fig. 4A). His-SIRT3 and Flag-ACSL4 plasmids were transfected into 293 T cells, and exogenous IP experiments also confirmed the interaction between ACSL4 and SIRT3 (Fig. 4B). Further, both endogenous and exogenous IF results (Figs. 4C and S6A) showed that ACSL4 and SIRT3 were co-localized and mainly localized in the cytoplasm. The interaction between ACSL4 and SIRT3 was analyzed by molecular docking, and the results indicated that ACSL4 interacted with SIRT3 (Fig. 4D). Subsequently, IP experiments showed that overexpression of SIRT3 inhibited the acetylation and protein expression of ACSL4 (Fig. 4E), while inhibition of SIRT3 with 3-TYP significantly enhanced the acetylation and protein expression of ACSL4 in NPC cells (Fig. 4F). It was reported that the mutation of 248 histidine (H) to tyrosine (Y) of SIRT3 can reduce its deacetylation activity [16]. Using SIRT3-H248Y mutant plasmid for IP experiments, it was found that the inactivation of deacetylase activity attenuated the inhibition of ACSL4 acetylation and protein expression by SIRT3 (Fig. 4G).

**Fig. 5:**
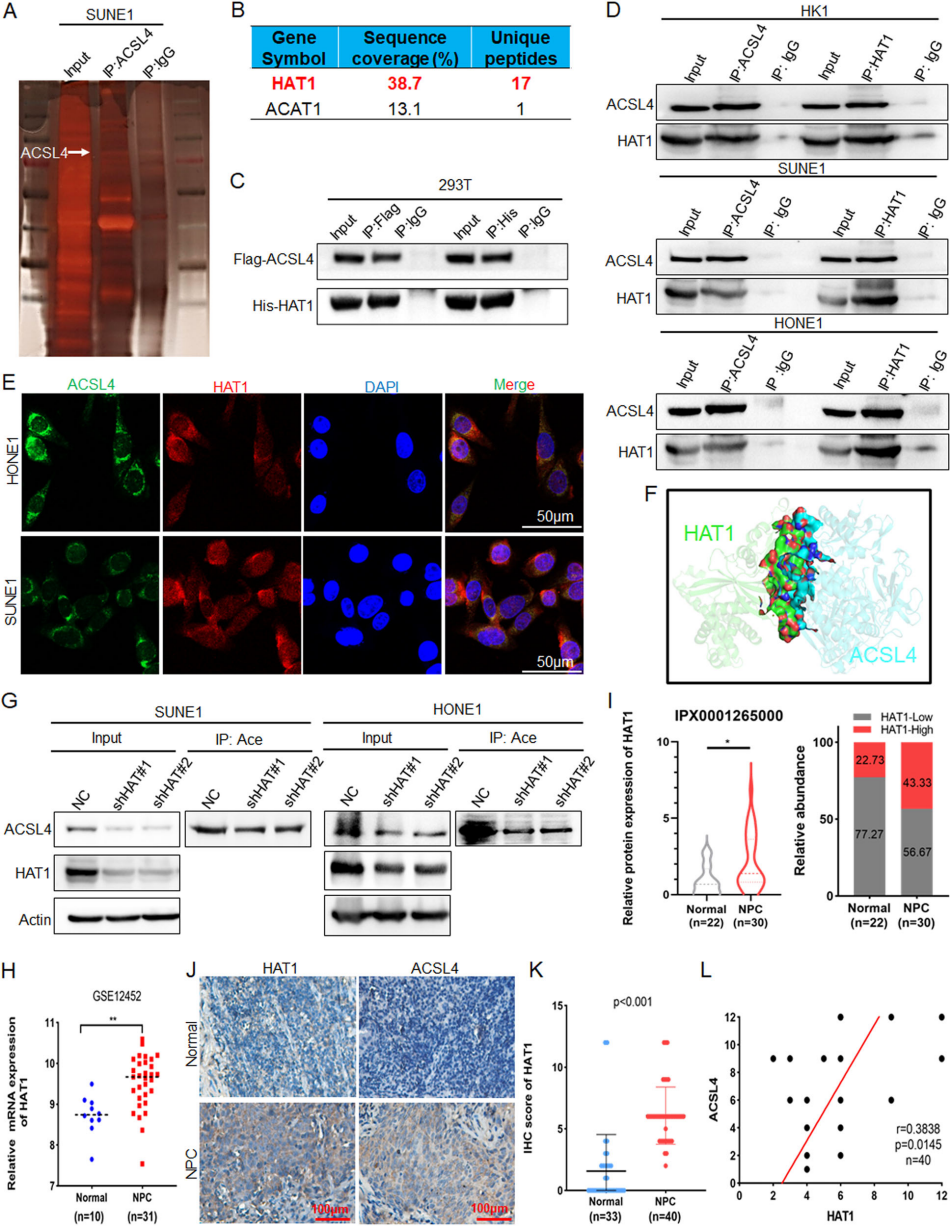
HAT1 directly mediates the acetylation of ACSL4. **A** Protein silver staining of IP by ACSL4 antibody enrichment. **B** Acetyltransferase in the protein mass spectrometry data. **C** His-HAT1 and Flag-ACSL4 plasmids were transfected into 293 T cells, and the interaction between ACSL4 and HAT1 was detected by IP. **D** IP was used to detect the interaction between ACSL4 and HAT1 in NPC cells. **E** The subcellular localization of ACSL4 and HAT1 was detected by IF. **F** Molecular docking analysis of the interaction between ACSL4 and HAT1. **G** HAT1 was knocked down in NPC cells, the acetylation of ACSL4 were detected by IP assay. **H** GSE12452 dataset was used to analyze the expression of HAT1. **I** The protein expression of HAT1 in NPC proteomics data. **J**, **K** The representative IHC images of HAT1 and ACSL4 in clinical samples and statistical analysis by IHC scores. **L** Correlation analysis for the expression of HAT1 and ACSL4 by IHC scores. Negative control (NC). The error line is expressed as mean ± SD. *p < 0.05, **p < 0.01.

To investigate the acetyltransferase that mediates the ACSL4 acetylation, IP experiments were performed to identify the ACSL4 antibody-enriched proteins by mass spectrometry (Fig. 5A), and found that HAT1 may be acetyltransferase of ACSL4 (Fig. 5B). Then His-HAT1 and Flag-ACSL4 plasmids were transfected into 293 T cells, and exogenous IP experiments confirmed that ACSL4 interacted with HAT1 (Fig. 5C). The endogenous interaction between ACSL4 and HAT1 was also confirmed by IP assay in NPC cells (Fig. 5D). Further endogenous and exogenous IF results (Figs. 5E and S6B) showed that ACSL4 and HAT1 were co-localized and mainly localized in the cytoplasm, and the data of molecular docking analysis also showed that ACSL4 interacted with HAT1 (Fig. 5F). Subsequently, IP experiments indicated that knockdown of HAT1 in NPC cells inhibited the acetylation of ACSL4 (Fig. 5G). Analysis of the GSE12452 dataset showed that HAT1 was highly expressed in NPC (Fig. 5H). Consistent with this, proteomics and IHC analysis of clinical samples of NPC showed that HAT1 was highly expressed in NPC (Fig. 5I–K). Correlation analysis displayed that HAT1 was positively correlated with ACSL4 (Fig. 5L). The above results indicated a direct involvement of HAT1 and SIRT3 in the acetylation and deacetylation process of ACSL4, respectively.

### Acetylation promotes the stability of ACSL4

**Fig. 6:**
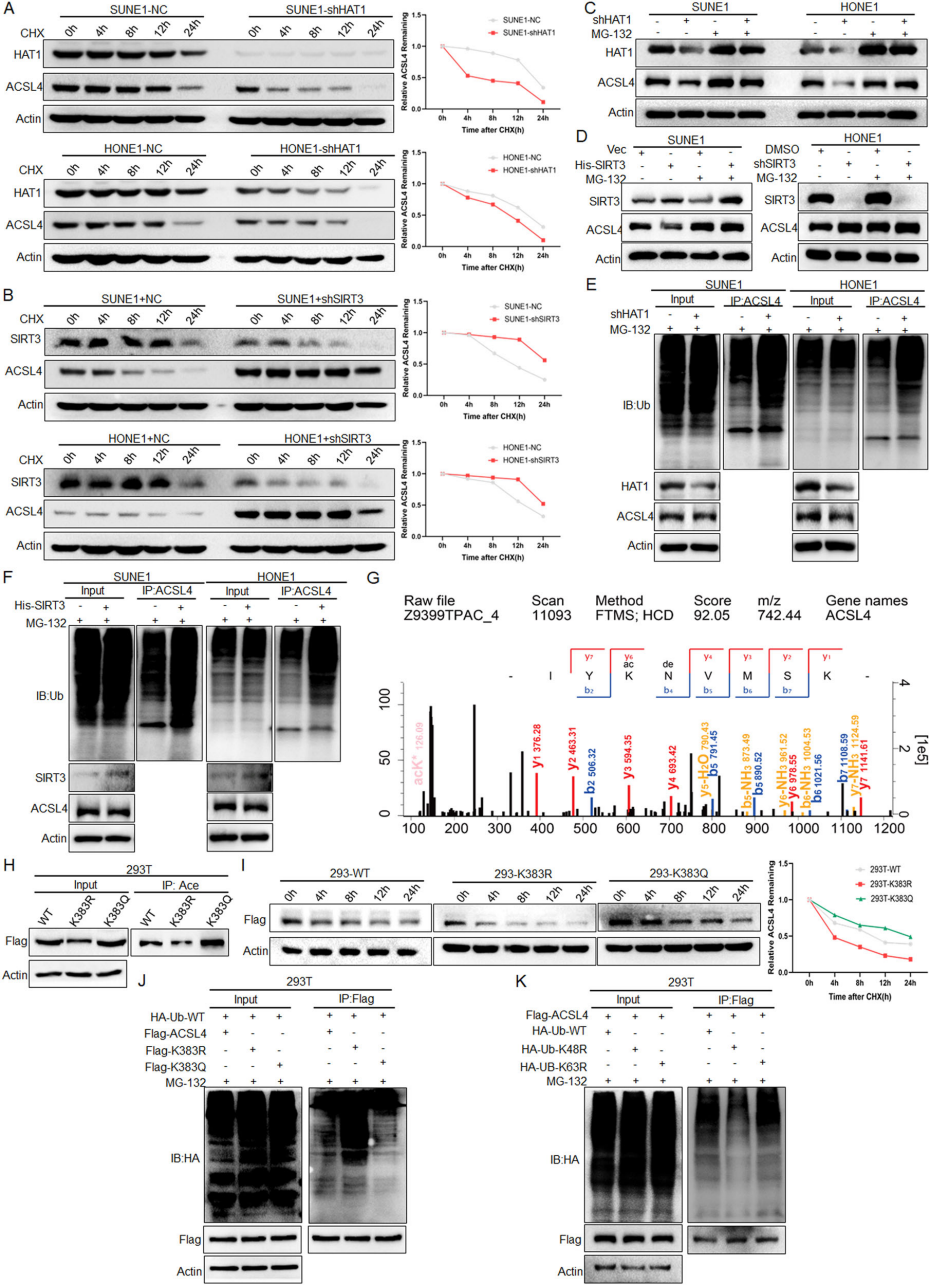
Acetylation promotes the stability of ACSL4. **A** HAT1 or **B** SIRT3 was knocked down in NPC cells, and the protein expression of ACSL4 was detected by western blot after CHX (10 μg/mL) treatment for different times (0, 4, 8, 12, 24 h). **C** HAT1 was knocked down and **D** SIRT3 was knocked down or overexpressed in NPC cells, the protein expression of ACSL4 was detected by western blot after treated with MG-132 (20 μM) for 24 h. **E** After knockdown of HAT1 and **F** overexpression of SIRT3 in NPC cells and treated with MG-132 (20 μM) for 10 h, the ubiquitination of ACSL4 was detected by IP assay. **G** ACSL4 acetylation site by mass spectrometry. After ACSL4-WT, ACSL4-K383R and ACSL4-K383Q plasmids were transfected into 293 T cells, **H** the acetylation of ACSL4 was detected by IP assay. **I** Treated with CHX (10 μg/mL) for different time (0, 4, 8, 12, 24 h), the protein expression of ACSL4 was detected by western blot. **J**, **K** Indicated plasmids were transfected into 293 T cells, then treated with MG-132 (20 μM) for 10 h, the ubiquitination of ACSL4 was detected by IP assay. Negative control (NC). Blank plasmid (vec).

To elucidate the mechanism of acetylation impacts ACSL4 expression, the protein stability experiment treated with CHX showed that knockdown of HAT1 reduced the protein stability of ACSL4 in NPC cells (Fig. 6A). Conversely, knockdown of SIRT3 significantly enhanced the protein stability of ACSL4 (Fig. 6B). In addition, LBH589 could reduce the stability of ACSL4 (Fig. S7). These data suggested that the acetylation of ACSL4 could enhance its protein stability. Subsequently, after being treated with the proteasome inhibitor MG-132, it was found that MG-132 treatment could rescue the ACSL4 downregulation caused by HAT1 knockdown (Fig. 6C) and high expression of SIRT3 (Fig. 6D), indicating that the protein stability of ACSL4 was mainly affected by the proteasome pathway. Further, knockdown of HAT1 (Fig. 6E) or overexpression of SIRT3 (Fig. 6F) in NPC cells, IP assay results showed that HAT1-mediated ACSL4 acetylation inhibited its ubiquitination, and SIRT3-mediated ACSL4 deacetylation promoted its ubiquitination. The previous acetylation proteomics results showed that the K383 of ACSL4 was its potential acetylation site (Fig. 6G). The Uniprot database analysis showed that K383 of ACSL4 exhibits high conserved among different species (Fig. S8A). Then the mutant plasmids of deacetylation (K383R) and acetylation (K383Q) of ACSL4 were constructed, and IP experiments further confirmed that K383 was its acetylation modification site (Fig. 6H). The spatial conformation of ACSL4 was demonstrated by Pymol software, it was found that the interaction between K383 and other amino acids increased after the acetylation of ACSL4 (Fig. S8B). Furthermore, the wild-type and mutant plasmids of ACSL4 were transfected into 293 T cells, and it was found that the K383R of ACSL4 could reduce the stability of ACSL4 (Fig. 6I). Importantly, the results of IP experiments showed that compared with the ACSL4-WT group, the acetylation of ACSL4 could inhibit its ubiquitination (Fig. 6J). Studies have shown that there are a variety of connection types of ubiquitination involved in the regulation of target proteins, of which K48 is mainly involved in the proteasome pathway degradation, while K63 is predominantly associated with signal transduction and protein stability [24]. Therefore, after the wild-type and mutant plasmids (K48 or K63) of ubiquitin were transfected into 293 T cells, the results indicated that the K48R inhibited the ubiquitination of ACSL4, while the K63R had no significant effect on the ubiquitination of ACSL4 (Fig. 6K). The above results revealed that K383 acetylation enhances ACSL4 protein stability by inhibiting its K48-linked ubiquitination degradation.

### K383 Acetylation inhibits FBXO10-mediated ubiquitination degradation of ACSL4

**Fig. 7:**
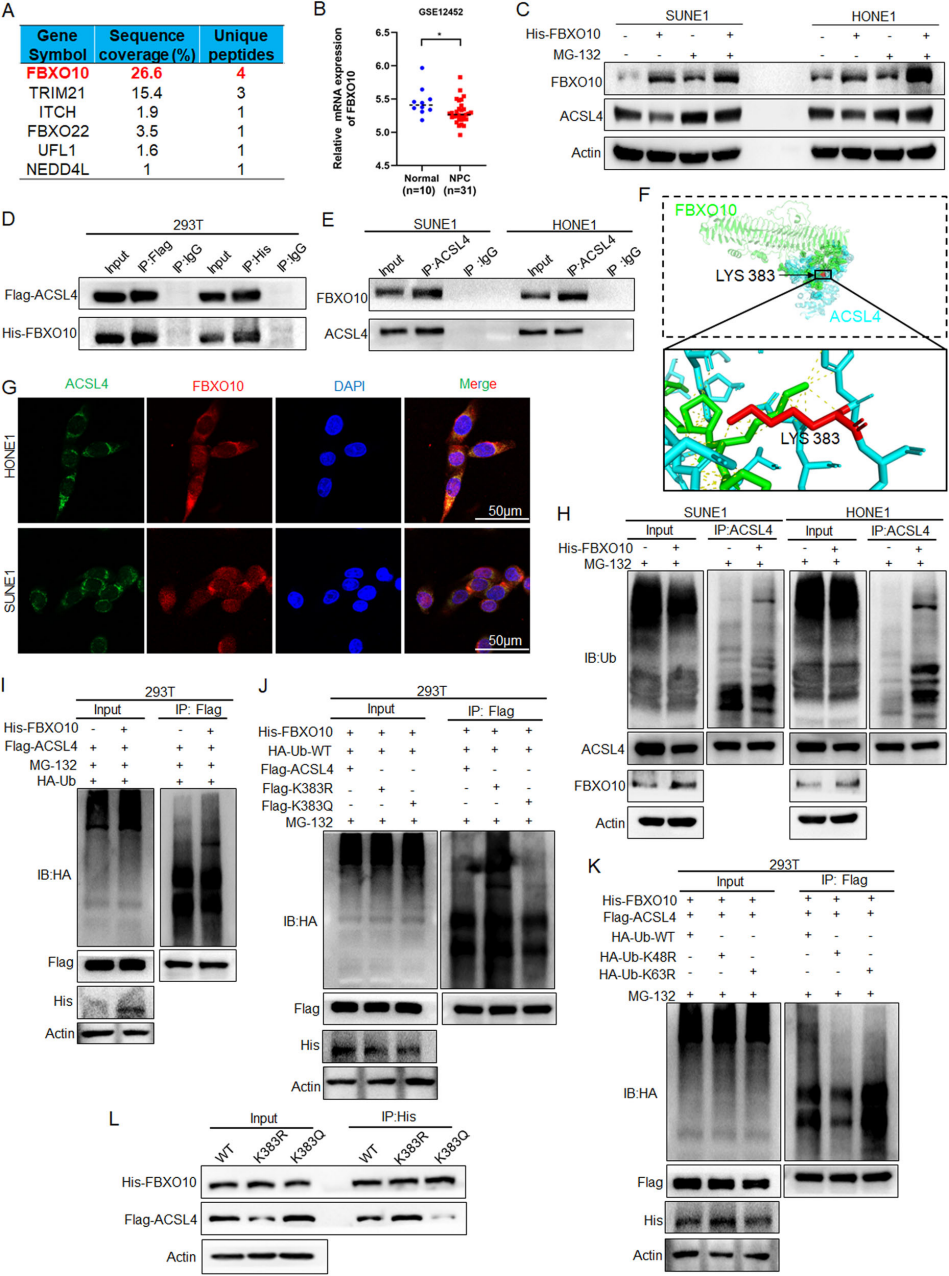
K383 acetylation inhibits FBXO10-mediated ubiquitination degradation of ACSL4. **A** E3 ligase in protein mass spectrometry data pulled down by ACSL4 antibody. **B** GSE12452 data was used to analyze the expression of FBXO10. **C** After overexpression of FBXO10 in NPC cells and treated with MG-132 (20 μM) for 24 h, the protein expression of ACSL4 was detected by western blot. **D** Flag-ACSL4 and His-FBXO10 plasmids were transfected into 293 T cells, and the interaction between ACSL4 and FBXO10 was detected by IP assay. **E** IP assay was used to detect the interaction between ACSL4 and FBXO10 in NPC cells. **F** Molecular docking analysis of the interaction between ACSL4 and FBXO10. **G** The subcellular localization of ACSL4 and FBXO10 was detected by IF assay. **H** After overexpression of FBXO10 and treated with MG-132 (20 μM) in NPC cells for 10 h, the ubiquitination of ACSL4 was detected by IP assay. **I** After transfection of Flag-ACSL4 plasmids and His-FBXO10 into 293 T cells and treated with MG-132 (20 μM) for 10 h, the ubiquitination of ACSL4 was detected by IP assay. **J**, **K** Indicated plasmids were transfected into 293 T cells, and treated with MG-132 (20 μM) for 10 h, the ubiquitination of ACSL4 was detected by IP assay. **L** Indicated plasmids were transfected into 293 T cells, the interaction between FBXO10 and ACSL4 was detected by IP assay. The error line is expressed as mean ± SD. *p < 0.05.

To explore which E3 ligase directly participates in ACSL4 ubiquitination, the E3 ligase in the previous mass spectrometry identification results of ACSL4 antibody enrichment was analyzed, and the highest enrichment was FBXO10 (Fig. 7A). And analysis of the GSE12452 dataset found that FBXO10 was lowly expressed in NPC (Fig. 7B). Further, overexpression of FBXO10 in NPC cells leaded to downregulation of ACSL4 protein, which could be inhibited by MG-132 treatment (Fig. 7C). Exogenous and endogenous interactions between ACSL4 and FBXO10 were determined by IP experiments in NPC cells and 293 T cells (Fig. 7D, E). And molecular docking analysis showed that ACSL4 interacted with FBXO10, and the K383 of ACSL4 was located in the domain of interaction between ACSL4 and FBXO10 (Fig. 7F). Further endogenous and exogenous IF results (Figs. 7G and S9) demonstrated that ACSL4 and FBXO10 were co-localized and mainly localized in the cytoplasm. Subsequently, IP experiments showed that overexpression of FBXO10 promoted the ubiquitination of ACSL4 (Fig. 7H). The same results were observed in the exogenous IP experiment in 293 T cells (Fig. 7I). To elucidate the effect of ACSL4 acetylation on FBXO10-mediated ubiquitination, IP experiments showed that the K383 acetylation of ACSL4 could inhibit FBXO10-mediated ubiquitination, while the deacetylation of ACSL4 could promote FBXO10-mediated ubiquitination (Fig. 7J). Moreover, IP experiments showed that the K48R could inhibit the ubiquitination of ACSL4 mediated by FBXO10, while the K63R had no significant effect (Fig. 7K). Importantly, it could be found that the K383 acetylation of ACSL4 inhibited the interaction between FBXO10 and ACSL4 (Fig. 7L). These results revealed that K383 acetylation of ACSL4 inhibits FBXO10-mediated K48-linked ubiquitination degradation by inhibiting the interaction between FBXO10 and ACSL4.

### K383 acetylation of ACSL4 promotes the malignant progression of NPC

**Fig. 8:**
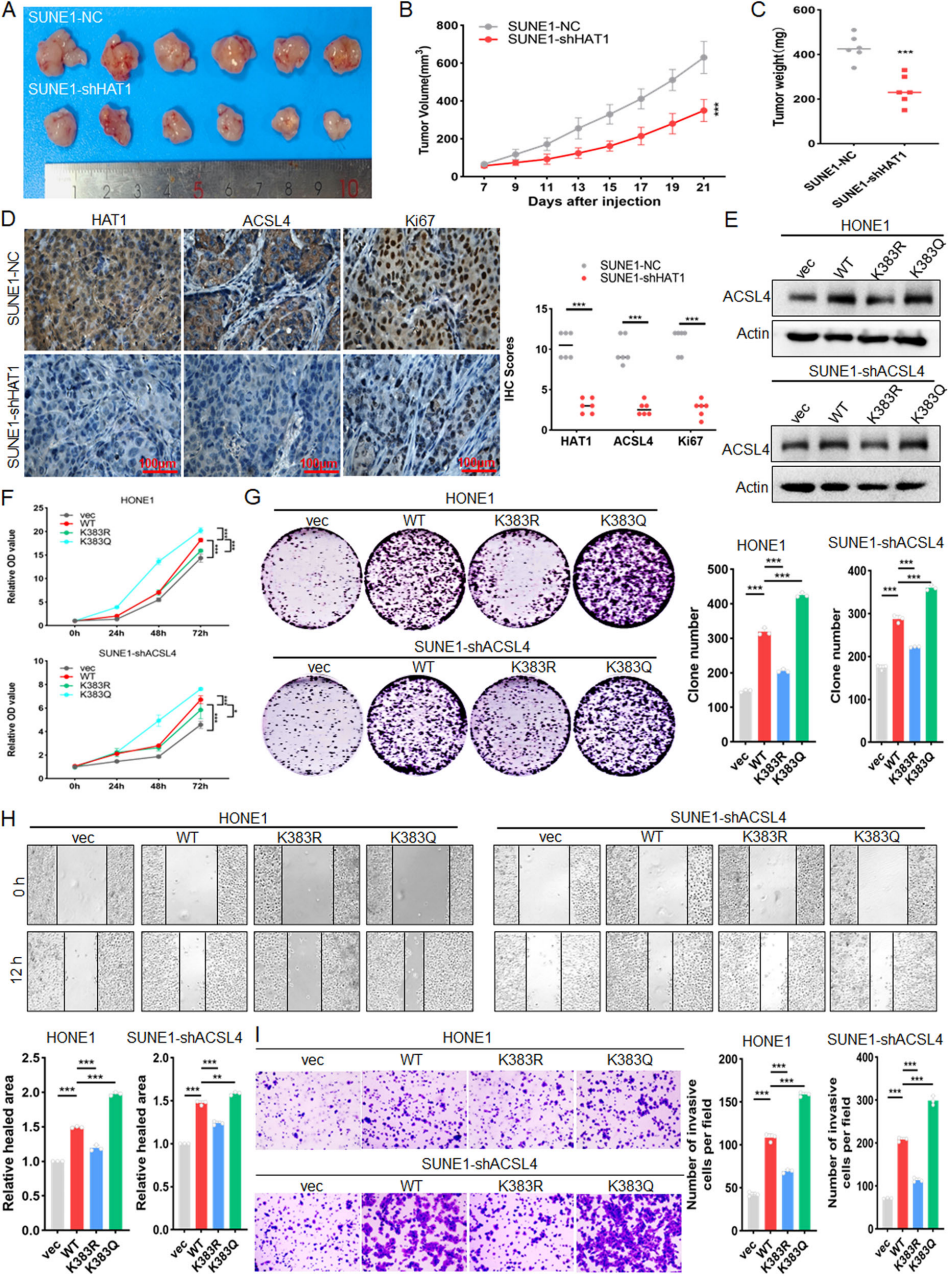
K383 acetylation of ACSL4 promotes the malignant progression of NPC. Subcutaneous injection of 2 × 10^6^ SUNE1-NC or SUNE1-shHAT1 cells into nude mice to construct a xenograft model (*n* = 6). **A** Tumor size, **B** tumor volume and **C** tumor weight was measured. **D** IHC was used to detect the expression of HAT1, ACSL4, and Ki67 in tumor tissues. SUNE1-shACSL4-vec, SUNE1-shACSL4-WT, SUNE1-shACSL4-K383R, SUNE1-shACSL4-K383Q cells and HONE1-vec, HONE1-WT, HONE1-K383R, HONE1-K383Q cells were used. **E** Western blot was used to detect the protein expression of ACSL4, **F** CCK8 and **G** colony formation assay was used to detect the cell proliferation. **H** Scratch assay and **I** Transwell assay were used to detect the cell migration and invasion. Blank plasmid (vec), negative control (NC). The error line is expressed as mean ± SD. *p < 0.05, **p < 0.01, ***p < 0.001.

To investigate the effect of K383 acetylation of ACSL4 on the malignant progression, we first explored the effect of HDAC2 in NPC. Tumor cells were treated with STA, CCK8 (Fig. S10A), colony formation (Fig. S10B), scratches (Fig. S10C), transwell (Fig. S10D) and xenograft experiments (Fig. 10E–G) showed that inhibition of HDAC2 by STA could weaken the malignant progression of NPC. Consistent with this, the results of IHC displayed that STA treatment could inhibit the expression of ACSL4 and Ki67 through increased SIRT3 (Fig. S10H). Meanwhile, after inhibition of SIRT3 with 3-TYP, and ACSL4 was further knocked down for rescue experiments (Fig. S11A), CCK8 (Fig. S11B), colony formation (Fig. S11C), scratch (Fig. S11D) and transwell (Fig. S11E) results revealed that 3-TYP could promote the malignant progression by upregulating the expression of ACSL4. Subsequently, SUNE1-NC and SUNE1-shHAT1 cells were used to establish xenograft models, and the results showed that knockdown of HAT1 could reduce the tumor growth (Fig. 8A–C). The results of IHC displayed that knockdown of HAT1 could inhibit the expression of ACSL4 and Ki67 (Fig. 8D). Further, overexpression of ACSL4 based on knocking down HAT1 for rescue experiments (Fig. S12A), CCK8 (Fig. S12B), colony formation (Fig. S12C), scratches (Fig. S12D), transwell (Fig. S12E) results showed that knocking down HAT1 reduced the malignant progression by inhibiting the expression of ACSL4. Similarly, stable NPC cell lines were constructed by expressing ACSL4 wild-type and K383 mutant plasmids in HONE1 or ACSL4 knockdown SUNE1 cells. The results of qPCR showed that the deacetylation of ACSL4 did not affect the mRNA level of ACSL4 (Fig. S13), but it could weaken its protein expression (Fig. 8E). The results of CCK8 and colony formation assays showed that K383R of ACSL4 could inhibit proliferation of NPC cells (Fig. 8F, G). And the results of scratch and transwell assays revealed that K383R of ACSL4 inhibited migration and invasion of NPC (Fig. 8H, I). The above results indicated that K383 acetylation of ACSL4 can promote the malignant progression of NPC.

### K383 acetylation of ACSL4 enhances radiosensitivity by inducing ferroptosis of NPC

**Fig. 9:**
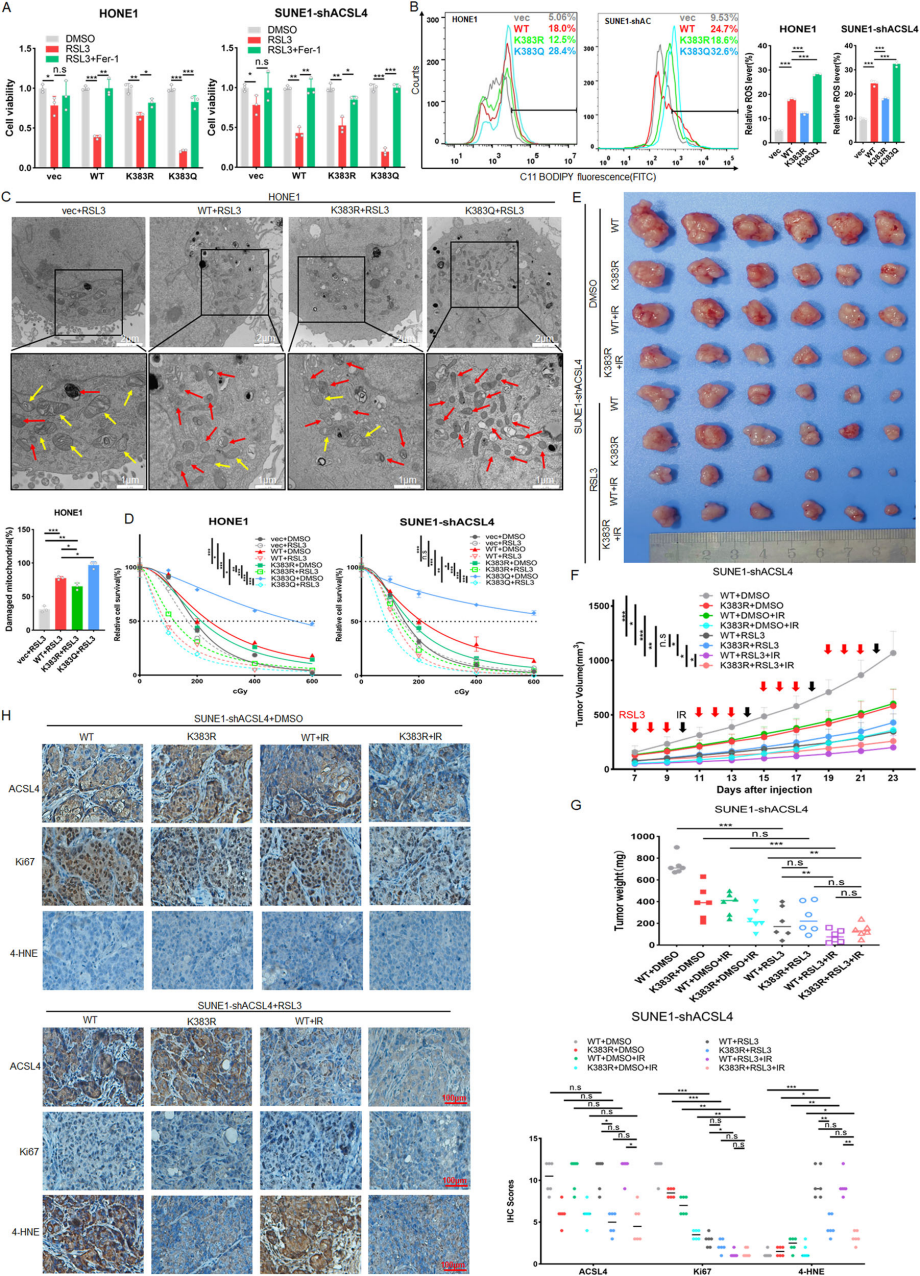
K383 acetylation of ACSL4 enhances radiosensitivity by inducing ferroptosis of NPC. The indicated stable cell lines were used. **A** CCK8 assay was used to detect the cell viability treated with RSL3 (5 μM) or with both RSL3 (5 μM) and Fer-1 (2 μM) for 24 h. **B** After treated with RSL3 (5 μM) for 24 h, lip-ROS levels were detected by flow cytometry. **C** Cells were treated with RSL3 (5 μM) for 24 h, the mitochondrial morphology was observed by TEM. **D** The radioresistance of NPC cells (3 × 10^3^ cells/well) was detected by colony formation assay under different doses (0, 1, 2, 4, 6 Gy) of IR and treatment with RSL3 (5 μM). 2 × 10^6^ SUNE1-shACSL4-WT or SUNE1-shACSL4-K383R cells were injected subcutaneously into nude mice and randomly divided into non-IR group and IR group, and then divided into DMSO treatment group and RSL3 treatment group (*n* = 6). **E** Tumor size, **F** tumor volume and **G** tumor weight was measured. **H** IHC was used to detect the expression of ACSL4, Ki67 and 4-HNE in tumor tissues. Blank plasmid (vec). The error line is expressed as mean ± SD. *p < 0.05, **p < 0.01, ***p < 0.001.

To evaluate the effect of K383 acetylation of ACSL4 on ferroptosis in NPC, the stable NPC cell lines of ACSL4 wild-type or K383 mutant treated with RSL3. The results of CCK8 assay showed that K383 deacetylation of ACSL4 could inhibit ferroptosis in NPC cells, while ACSL4 acetylation could promote ferroptosis, which could be inhibited after treated with Fer-1 (Fig. 9A). Meanwhile, under the treatment of RSL3, the results of flow cytometry analysis displayed that the K383 deacetylation of ACSL4 could inhibit the lip-ROS level of NPC cells (Fig. 9B). The ultrastructural changes of mitochondria were further analyzed by TEM. Compared with the blank plasmid group, after HONE1-ACSL4-WT cells were treated with RSL3, a lot of mitochondria shrank and the number of cristae decreased significantly, while the K383R of ACSL4 could weaken this effect (Fig. 9C), indicating that deacetylated ACSL4 could inhibit ferroptosis in NPC cells. Subsequently, we evaluated the effect of ferroptosis induced by different acetylation states of ACSL4 on radiosensitivity. The results showed that the acetylation of ACSL4 enhanced the radioresistance of NPC cells. Simultaneously, it also increased RSL3-induced ferroptosis and further promoted radiosensitivity (Figs. 9D and S14). Further, the results of in vivo experiments displayed that the K383R of ACSL4 reduced RSL3-induced ferroptosis and further weakened the radioresistance of NPC (Fig. 9E–G). IHC analysis showed that the K383R of ACSL4 reduced the positive rate of 4-HNE and Ki67 in tumor cells (Fig. 9H). The above results indicated that K383 acetylation of ACSL4 enhances radiosensitivity by promoting ferroptosis in NPC.

## Discussion

ACSL4 plays a promoter or suppressor role in tumors depending on the specific cancer type and tissue environment [25]. ACSL4 inhibits cell proliferation and migration through the downregulation of focal adhesion kinase FAK and the upregulation of p21 expressions in gastric cancer [26]. However, it exerts oncogenic functions in most cases. ACSL4 not only promotes the intracellular lipogenesis and lipid droplets accumulation but also enhances fatty acid oxidation and adenosine triphosphate production by upregulating CPT1A to drive breast cancer metastasis [27]. In hepatocellular carcinoma, ACSL4 upregulates the main lipogenic regulator SREBP1 through c-Myc, leading to the accumulation of intracellular triglycerides, cholesterol and lipid droplets, thereby promoting malignant tumor progression [28]. ACSL4 promotes the malignant progression by upregulating the overall protein myristoylation in estrogen receptor (AR)-dependent prostate cancer cells [29]. In recent years, studies have found that ACSL4 can positively regulate ferroptosis by catalyzing fatty acid oxidation to produce acyl-CoA [27], which can be used to inhibit malignant progression of tumors. For example, ACSL4 inhibits glioma proliferation by activating ferroptosis in tumor cells [30]. In colorectal cancer, ACSL4 promotes lipid peroxidation and ferroptosis in tumor cells and confers sensitivity to oxaliplatin [31]. Meaningfully, plenty of studies have found that tumor cells with high malignancy tend to have higher ferroptosis sensitivity [32, 33]. Adenosine cyclase ADCY10 is highly expressed in lung adenocarcinoma, and it promotes the development of lung adenocarcinoma and also induces ferroptosis sensitivity of tumor cells [34]. O-GlcNAc transferase (OGT) is upregulated in clear cell renal cell carcinoma, and promotes proliferation, colony formation and invasion of tumor cells. Meanwhile, OGT enhances the sensitivity of tumor cells to ferroptosis [35]. In this study, we determined that ACSL4 is highly expressed in NPC and plays a double-edged sword role in NPC. On the one hand, ACSL4 could promote the malignant progress of tumors; on the other hand, it enhances radiosensitivity by endowing NPC cells with ferroptosis-sensitive properties.

Studies have shown that IR can induce the expression of ACSL4, thereby triggering ferroptosis in tumor cells [36]. Therefore, as shown in Fig. S15A, we treated NPC cells with IR and the results showed that IR treatment upregulated the protein expression of ACSL4 but did not affect its acetylation level. The data also demonstrated that IR treatment did not affect the protein expression of HAT1, HDAC2, or SIRT3, indicating that the IR-induced increase of ACSL4 expression is not regulated through acetylation modification (Fig. S15B). Due to the high expression of ACSL4 mediated by acetylation in NPC cells, combined with clinical radiotherapy, IR induced ACSL4 expression will further enhance the ferroptosis sensitivity of NPC, thereby helping to improve the radiation therapy effect of NPC.

Research has found that the expression of ASCL4 in tumor cells is regulated by multiple mechanisms. Transcription factor ZEB2 activates ACSL4 expression by directly binding to the ACSL4 promoter, thereby promoting cellular lipid storage and fatty acid oxidation to drive breast cancer metastasis [27]. In renal cell carcinoma cells, receptor protein AIM2 promotes FOXO3a phosphorylation and proteasome degradation, thereby abating its transcriptional effect on ACSL4 and inhibiting ferroptosis [37]. In breast cancer, PKCβII activates ACSL4 through phosphorylation to increase ferroptosis-related lipid peroxidation. Attenuation of the PKCβII-ACSL4 pathway effectively blocks ferroptosis in vitro and impairs ferroptosis-associated cancer immunotherapy [38]. In colorectal cancer, coactivator-associated arginine methyltransferase CARM1 methylates arginine 339 (R339) of ACSL4, promotes the binding of RNF25 to ACSL4 and its ubiquitination, inhibits ferroptosis of tumor cells, and effectively attenuates ferroptosis-related cancer immunotherapy [39]. In current study, for the first time, we elucidated the importance of acetylation modification of ACSL4 on its expression regulation in NPC, and clarified that HAT1 and SIRT3 mediates the K383 dynamic acetylation of ACSL4, and the acetylation of ACSL4 weakens its interaction with FBXO10, thereby inhibiting FBXO10-mediated K48-linked ubiquitination and degradation.

In the regulation of protein enzymatic acetylation, the redundant functions of acetyltransferases or deacetylases have been reported. For instance, there is low-level redundancy of acetyltransferases function in the MYST family (HAT1-3) [40]. HDAC1 and HDAC2 are two highly homologous Class I HDACs and display compensatory or specific roles in different cell types or in response to different stimuli and signaling pathways. In most mouse knockout studies, deletion of both enzymes is required to produce a substantial phenotype [41]. Another example is SIRT3 and SIRT5, which may compensate each other considering that they share subcellular location and targets [42]. In our study, we identified SIRT3 as the indispensable deacetylase to ensure ACSL4 deacetylation in NPC cells, and HDAC2 upregulates ACSL4 acetylation by inhibiting SIRT3 transcription. During this process, no compensatory effects of other deacetylases were observed by combining various broad-spectrum or specific deacetylase inhibitors with shRNA screening. In addition, based on protein data of IP/MS, acetyltransferase HAT1 and ACAT1 were found to potentially interact with ACSL4. We further validated that HAT1 is the acetyltransferase for ACSL4, however, ACAT1 might also be involved in the acetylation modification of ACSL4, which requires further analysis. Additionally, acetyl-CoA mediated protein non-enzymatic acetylation is another mechanism, however, it is still not fully clear in what conditions non-enzymatic acylation is regulated and what its functions are [11]. Regarding the ACSL4 acetylation, further exploration is needed to determine whether there is non-enzymatic acetylation compensation in the absence of HAT1 or HDAC2/SIRT3.

Deacetylase inhibitors are an attractive strategy for reversing abnormal epigenetic changes associated with cancer treatment [43]. Several HDAC inhibitor drugs, such as vorinostat, romidepsin, panobinostat and Belinostat, have been approved by the FDA [44]. In addition, many specific deacetylase inhibitors are applied in clinical research. The STA inhibits the HDAC2/β-catenin signaling pathway and suppresses the migration and invasion of pancreatic cancer cells [45]. The inhibitory effect of 3-TYP can restore the anti-proliferation and migration of atractylon in glioblastoma cells [46], which is consistent with our results. STA can inhibit the malignant progression of NPC, while 3-TYP can promote the malignant progression of NPC. This indicates that not all HDAC inhibitor can exert anti-tumor effects, and the inhibitor need to be selected based on the expression and function of their target molecules in different tumors.

**Fig. 10:**
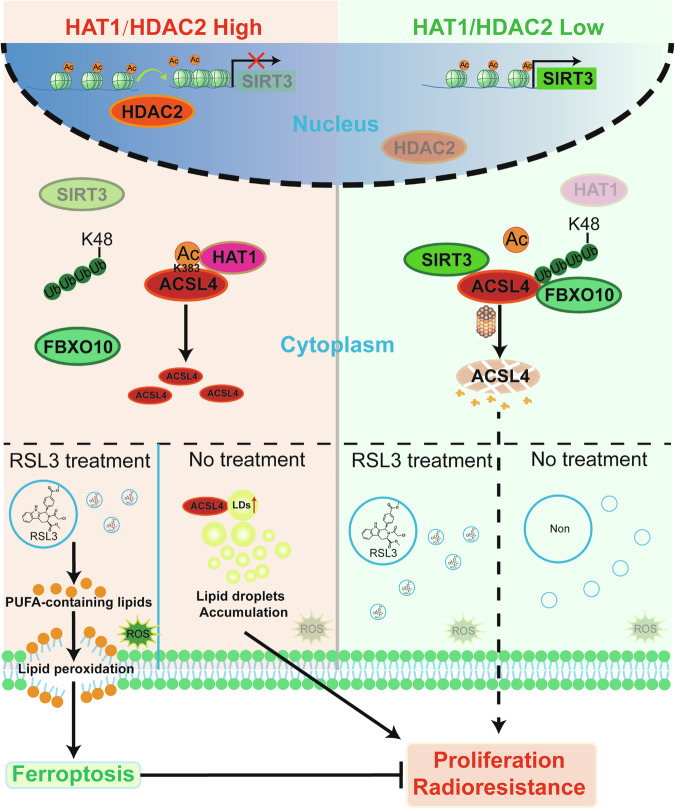
Graphic overview. Schematic of the model for HAT1/HDAC2 regulating ACSL4 acetylation and protein expression.

In summary, we found that HDAC2 and HAT1 are highly expressed in NPC. HDAC2 could reduce the transcription of SIRT3, which mediated the deacetylation K383 of ACSL4, meanwhile, HAT1 directly promoted the acetylation of ACSL4. The acetylation of ACSL4 could inhibit FBXO10-mediated K48-linked ubiquitination, resulting in enhanced protein stability of ACSL4, thereby promoting the double-edged sword effect: malignant progression and ferroptosis of NPC cells (Fig. 10).

## Supplementary information

Supplementary Materials

Original Data

## Data Availability

Source data and reagents are available from the corresponding author upon reasonable request.
