# Supplementary material for: HAT1/HDAC2 mediated ACSL4 acetylation confers radiosensitivity by inducing ferroptosis in nasopharyngeal carcinoma
Source: Cell Death Dis. 2025 Mar 6;16(1):160. doi: 10.1038/s41419-025-07477-4 (PMC11885570; doi:10.1038/s41419-025-07477-4)
Supplement: Supplementary file 1 — Supplementary Materials [file 41419_2025_7477_MOESM1_ESM.docx]

**Supplementary Materials**

**HAT1/HDAC2 mediated ACSL4 acetylation** **confers** **radiosensitivity by** **inducing ferroptosis in nasopharyngeal carcinoma**

Peijun Zhou, Xingzhi Peng, Kun zhang, Jin Cheng, Min Tang, Lin Shen, Qin Zhou, Dan Li, Lifang Yang

**Content**

● **Supplementary Figure S1.** Differentially modified and differentially expressed proteins were enriched in the ferroptosis pathway

● **Supplementary Figure S2.** The high acetylation of ACSL4 is associated with HDAC2 in NPC

● **Supplementary Figure S3.** HDAC2/SIRT3 mediates the acetylation and high expression of ACSL4 in NPC

● **Supplementary Figure S4.** ACSL4 promotes the malignant progression of NPC

● **Supplementary Figure S5.** ACSL4 promotes ferroptosis in NPC cells

● **Supplementary Figure S6.** ACSL4 exhibits subcellular colocalization with SIRT3 and HAT1, respectively

**● Supplementary Figure S7.** LBH589 reduces the stability of ACSL4

**● Supplementary Figure S8.** The K383 of ACSL4 is highly conserved

**● Supplementary Figure S9. ACSL4 exhibits subcellular colocalization with FBXO10**

**● Supplementary Figure S10.** Inhibition of HDAC2 hinders the malignant progression of NPC

**● Supplementary Figure S11.** SIRT3 inhibits ACSL4-mediated malignant progression of NPC cells

**● Supplementary Figure S12.** HAT1 promotes ACSL4-mediated malignant progression of NPC cells

**● Supplementary Figure S13.** The K383 mutation of ACSL4 does not affect its mRNA level

**● Supplementary Figure S14.** K383 acetylation of ACSL4 enhances radiosensitivity of NPC cells by inducing ferroptosis

**● Supplementary Figure S15.** IR can increase the protein expression of ACSL4 without affecting its acetylation

● **Supplementary Table S1.** Primers for homologous recombination

● **Supplementary Table S2.** Target sequences for RNA interference

● **Supplementary Table S3.** Primers for qPCR

● **Supplementary Table S4.** Clinical characteristics of NPC patients (pathological sections)

**Supplementary Figures**

**
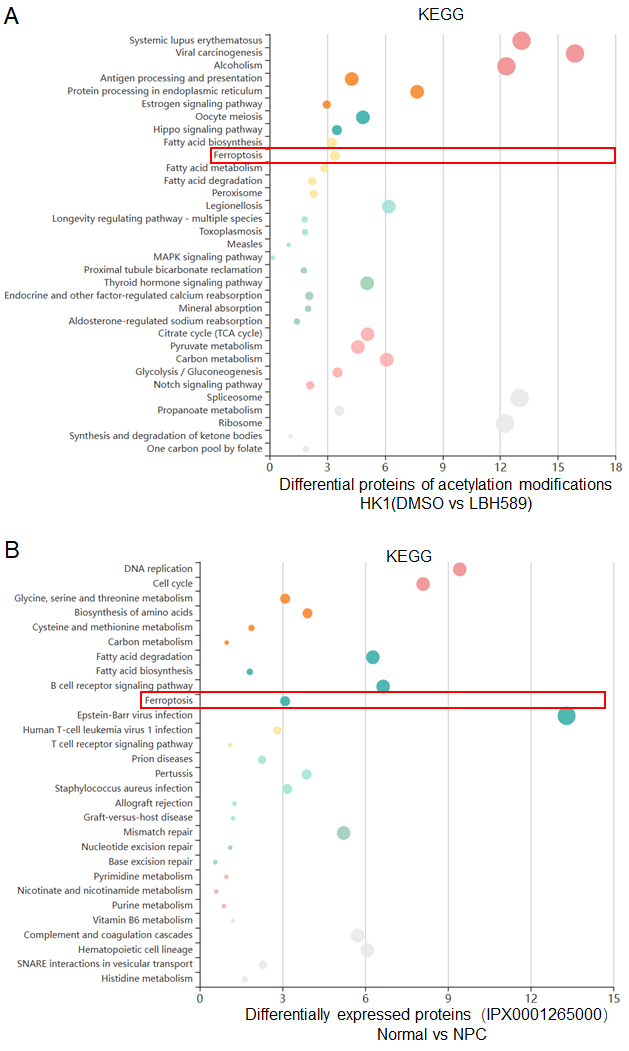
**

**Supplementary Fig.S1** **Differentially acetylated and differentially expressed proteins were enriched in the ferroptosis pathway.** (A) KEGG analysis of differential acetylated proteins from LS-MS/MS results. (B) KEGG analysis of differentially expressed proteins from IPX0001265000 database.


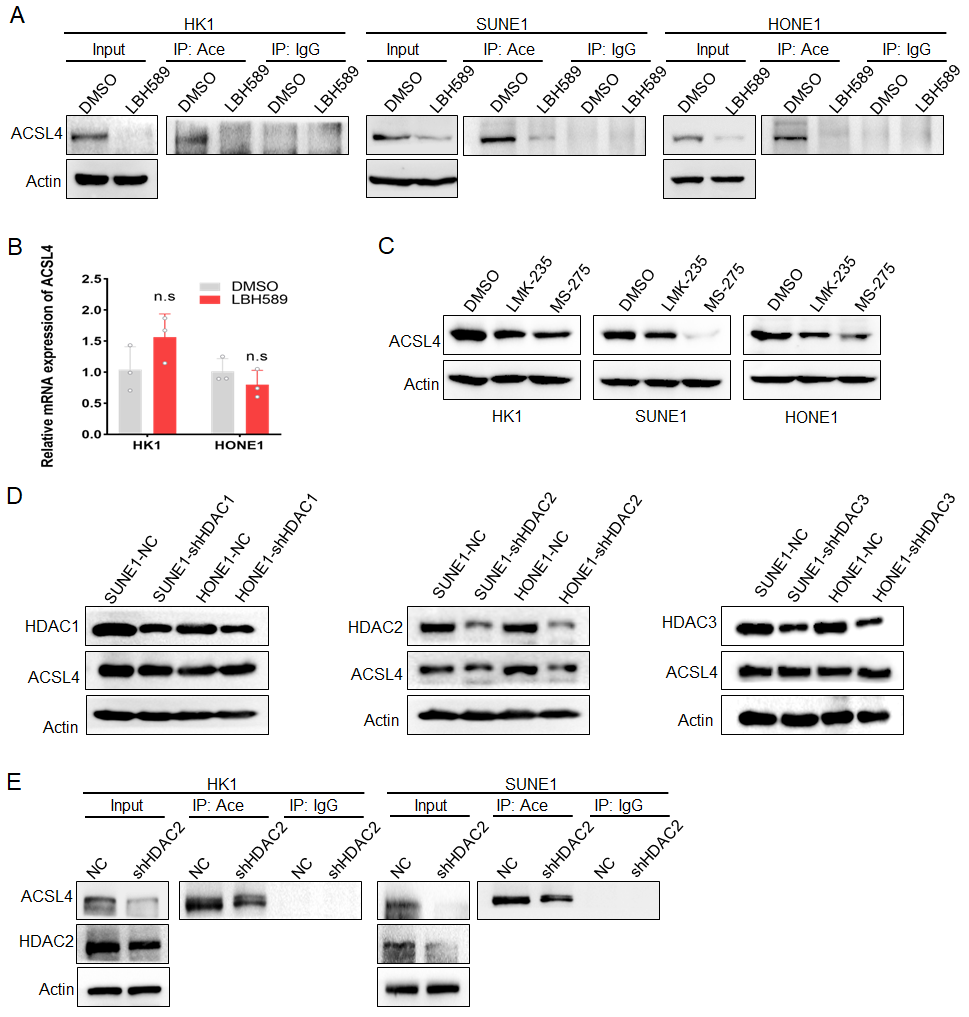


**Supplementary Fig.S2 The high acetylation of ACSL4 is associated with HDAC2 in NPC.** (A) NPC cells were treated with LBH589 (100nM) for 24 hours, and the acetylation of ACSL4 was detected by IP assay. After knocking down HDAC2 in NPC cells. (B) HK1 and HONE1 cells were treated with LBH589 (100nM) for 24 hours, and the mRNA level of ACSL4 was detected by qPCR. (C) NPC cells were treated with MS-235 (1μM) and LMK-275 (1μM) for 24 hours, and the protein expression of ACSL4 was detected by western blot. (D) After shHDAC1, shHDAC2 and shHDAC3 plasmids were transfected into NPC cells respectively, the protein expression of HDAC1, HDAC2, HDAC3 and ACSL4 were detected by western blot. (E) The acetylation of ACSL4 was detected by IP assay. The error line is expressed as mean ± SD. n.s p>0.05.


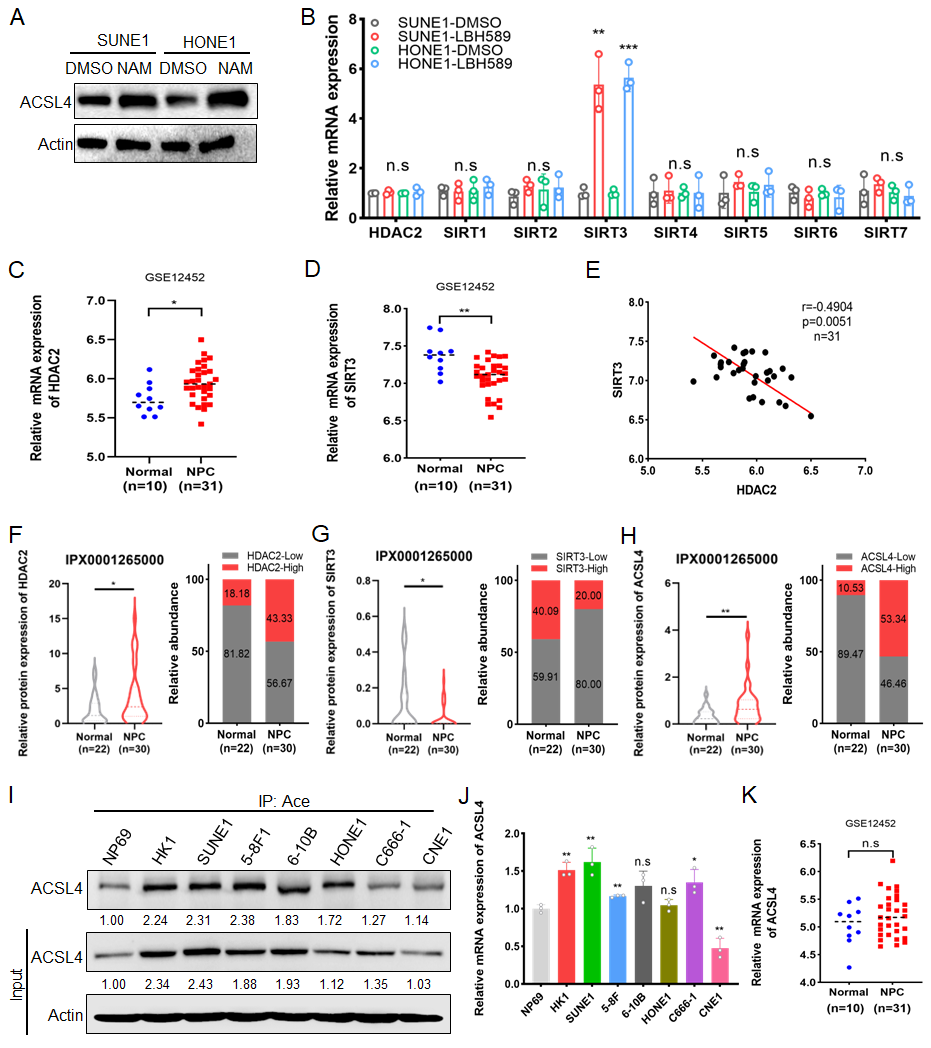


**Supplementary Fig.S****3 HDAC2/SIRT3 mediates the acetylation and high expression of ACSL4 in NPC.** (A) NPC cells were treated with NAM (100μM) for 24 hours, and the protein level of ACSL4 was detected by western blot. (B) NPC cells were treated with LBH589 (100nM) for 24 hours, the mRNA levels of HDAC2 and SIRT1-7 were detected by qPCR. GSE12452 dataset was used to analyze the mRNA level of (C) HDAC2, (D) SIRT3 and (E) correlation analysis of HDAC2 and SIRT3. Proteomics data analysis for the expression level of (F) HDAC2, (G) SIRT3, and (H) ACSL4. (I) NPC cells and normal nasopharyngeal epithelial cells were used to detect acetylation of ACSL4 by IP experiments. (J) The mRNA level of ACSL4 was analyzed by qPCR in NPC cells. (K) GSE12452 dataset was used to analyze the mRNA level of ACSL4. Negative control (NC). The error line is expressed as mean ± SD. n.s p>0.05, *p<0.05, **p<0.01, ***p<0.001.

**
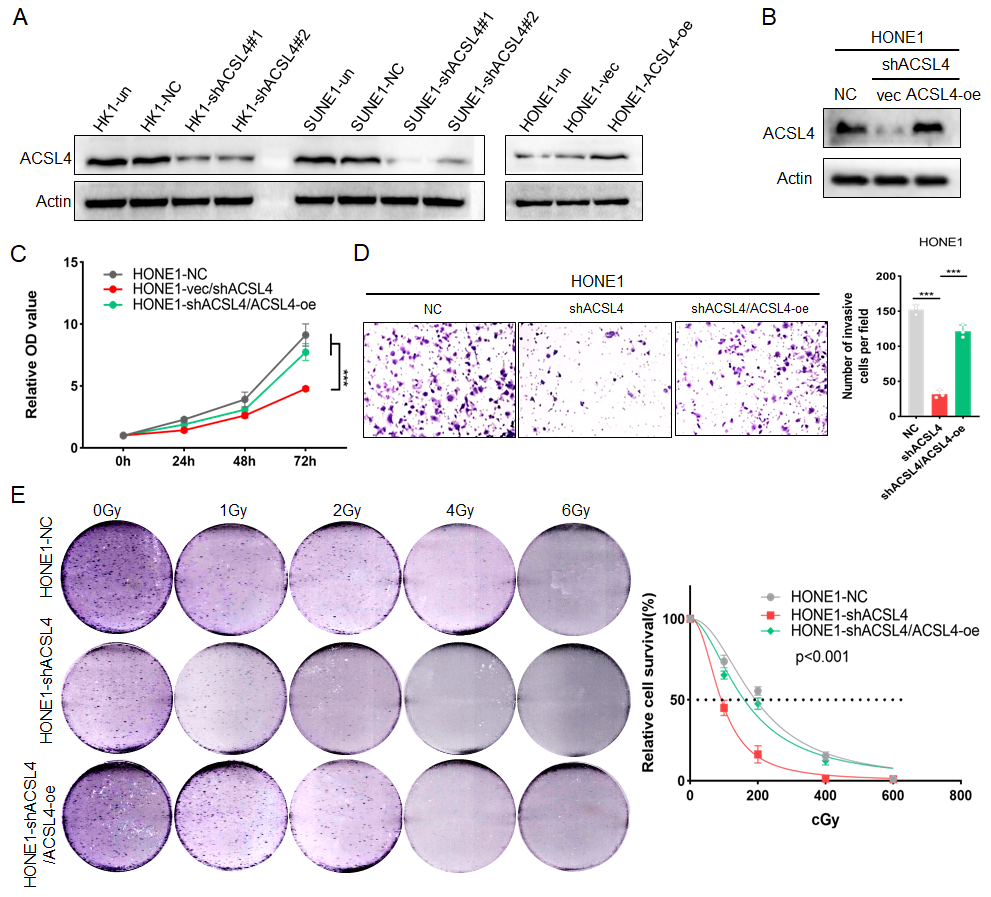
**

**Supplementary Fig.S4 ACSL4 promotes the malignant progression of NPC.** (A) HK1-NC, HK1-shACSL4#1, HK1-shACSL4#2, SUNE1-NC, SUNE1-shACSL4#1, SUNE1-shACSL4#2 and HONE1-vec, HONE1-ACSL4-oe cell lines were constructed, and the protein expression of ACSL4 was detected by western blot. (B) ACSL4 was overexpressed in HONE1-shACSL4 cells, and the protein levels of ACSL4 were detected by Western blot. (C) CCK8 assay was used to detect the cell proliferation. (D) Transwell assay was used to detect the cell invasion. (E) The radioresistance of HONE1 cells (2×10^3^ cells/well) was detected by colony formation assay under different doses (0, 1, 2, 4, 6Gy) of IR. Untreated group (un), negative control (NC). The error line is expressed as mean ± SD. ***p<0.001.


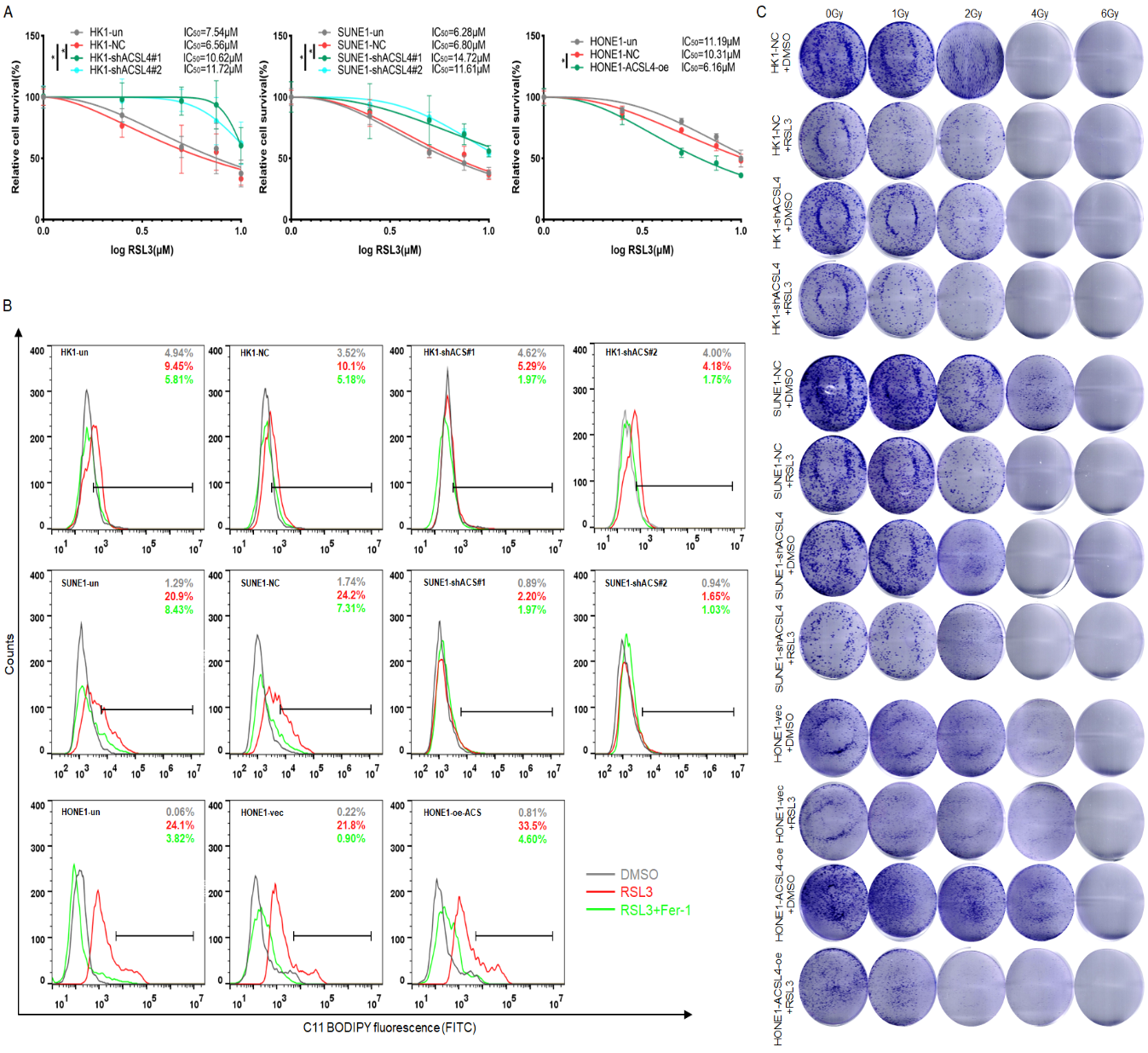


**Supplementary Fig.S5. ACSL4 induces ferroptosis in NPC cells.** HK1, HK1-NC, HK1-shACSL4#1, HK1-shACSL4#2, SUNE1, SUNE1-NC, SUNE1-shACSL4#1, SUNE1-shACSL4#2 and HONE1, HONE1-vec, HONE1-ACSL4-oe cell lines were used, (A) CCK8 assay was used to detect the IC50 value of cells treated with different concentrations (0,2.5,5,7.5,10μM) of RSL3for 24 hours. (B) Flow cytometry was used to analyze lip-ROS levels treated with RSL3 (5μM) or with both RSL3 (5μM) and Fer-1 (2μM) for 24 hours. (C) The radioresistance of NPC cells (3×10^3^ cells/well) was detected by colony formation assay under different doses (0, 1, 2, 4, 6Gy) of IR. Untreated group (un), negative control (NC), blank plasmid (vec). The error line is expressed as mean ± SD. *p<0.05.


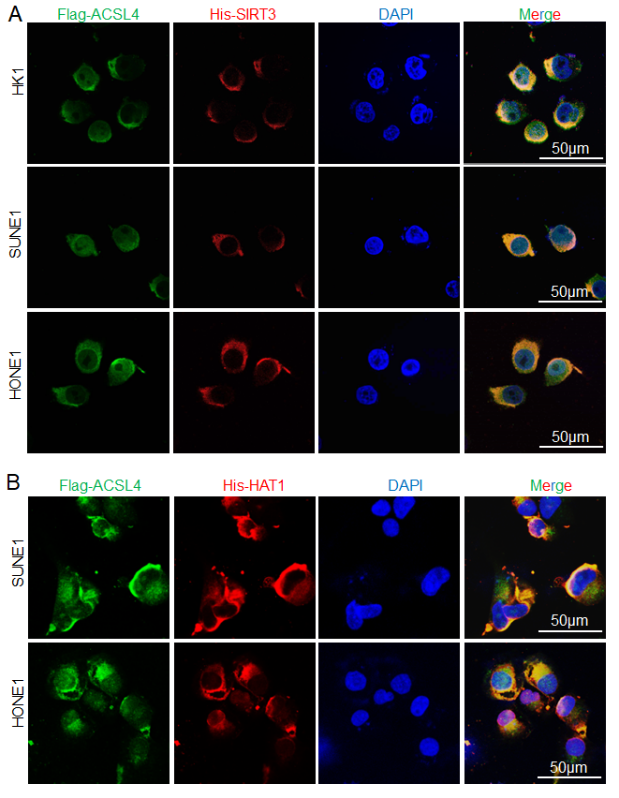


**Supplementary Fig.S6 ACSL4 exhibits subcellular colocalization with SIRT3 and HAT1, respectively.** Indicated plasmids were transfected into NPC cells. (A) The subcellular localization of ACSL4 and SIRT3 was detected by IF. (B) The subcellular localization of ACSL4 and HAT1 was detected by IF.


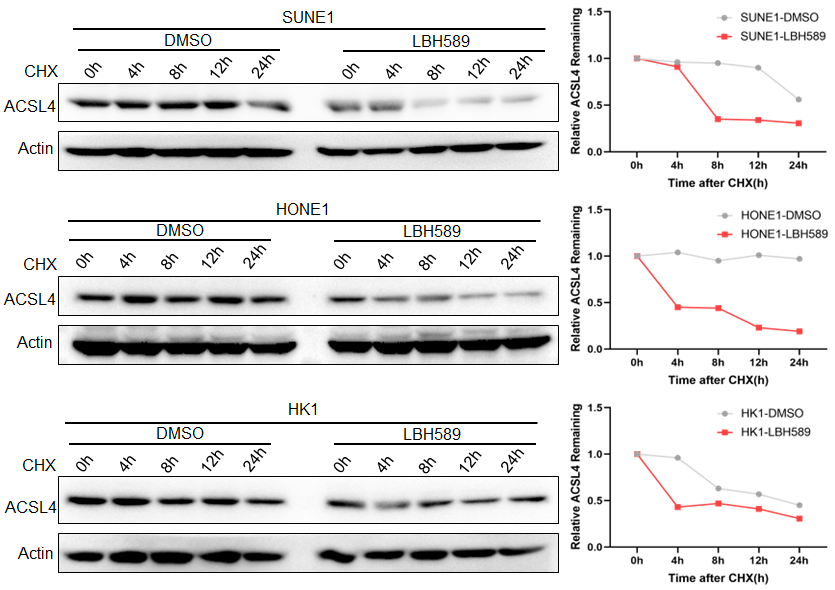


**Supplementary Fig.S7 LBH589 reduces the stability of ACSL4.** After NPC cells were treated with LBH589 (100nm) for 24 hours, then treated with CHX (10μg/mL) for different time (0, 4, 8, 12, 24 hours), the protein expression of ACSL4 was detected by western blot.


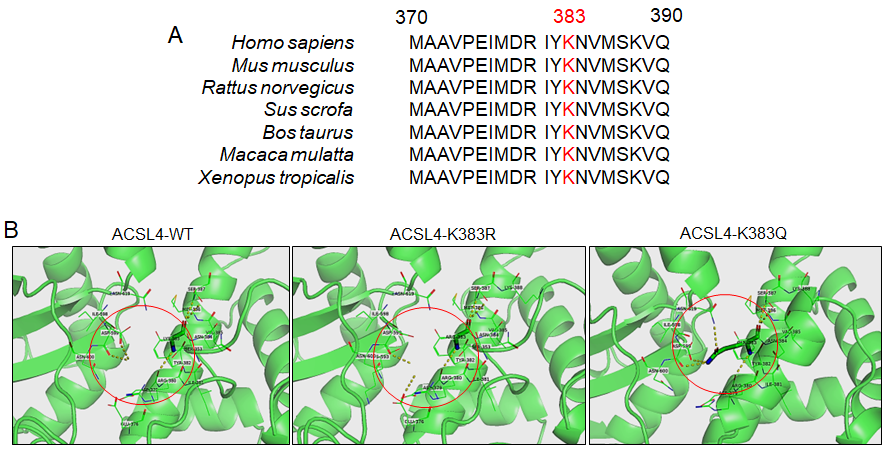


**Supplementary Fig.S8 The K383 of ACSL4 is highly conserved.** (A) The Uniprot database was used to analyze the species conservation of K383 in ACSL4. (B) Pymol software was used to display the spatial conformation of ACSL4 wild type and site mutation.


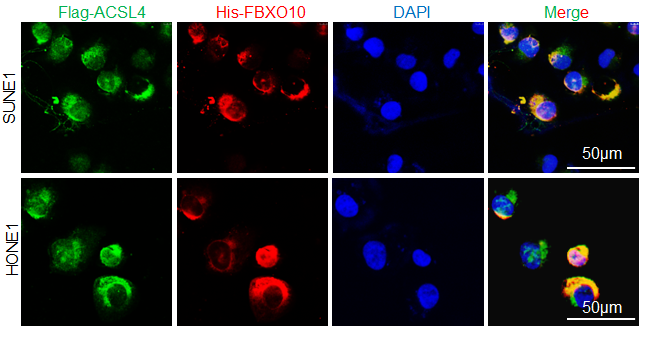


**Supplementary Figure S9. ACSL4 exhibits subcellular colocalization with FBXO10.** Indicated plasmids were transfected into NPC cells, and the subcellular localization of ACSL4 and FBXO10 was detected by IF assay.


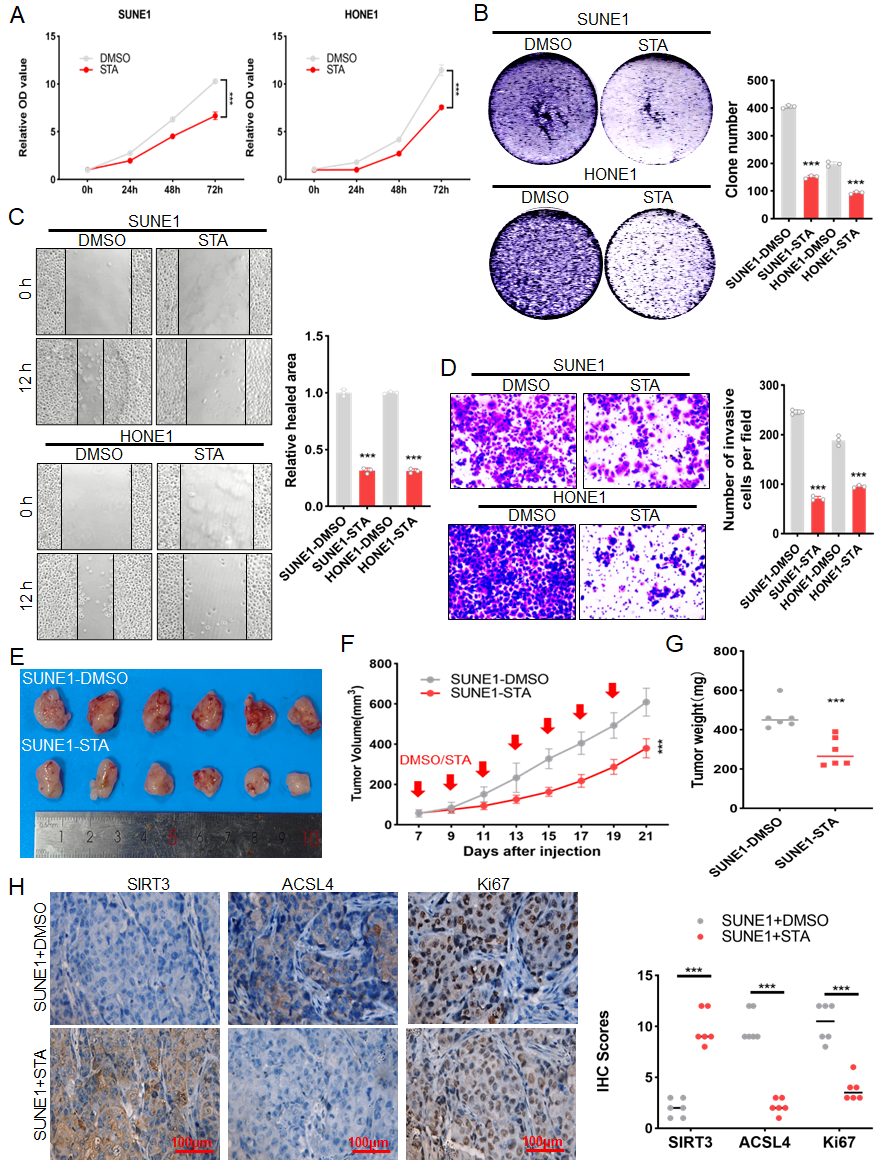


**Supplementary Fig.S10** **Inhibition of HDAC2 hinders the malignant progression of NPC.** SUNE1 and HONE1 cells were treated with STA (20μM). (A) CCK8 and (B) colony formation assay were used to analyze cell proliferation. (C) Scratch assay was used to detect the cell migration. (D) Transwell assay was used to detect the cell invasion. 5×10^6^ SUNE1 cells were subcutaneously injected into nude mice to construct a xenograft model. The mice were randomly divided into DMSO group and STA group [intraperitoneal injection of STA (50mg/kg) dissolved in 100μL DMSO, once every other day] (n=6). (E) tumor size, (F) tumor volume and (G) tumor weight were measured. (H) IHC was used to analyze the expression of SIRT3, ACSL4 and Ki67 in tumor tissues. The error line is expressed as mean ± SD. *** p < 0.001.


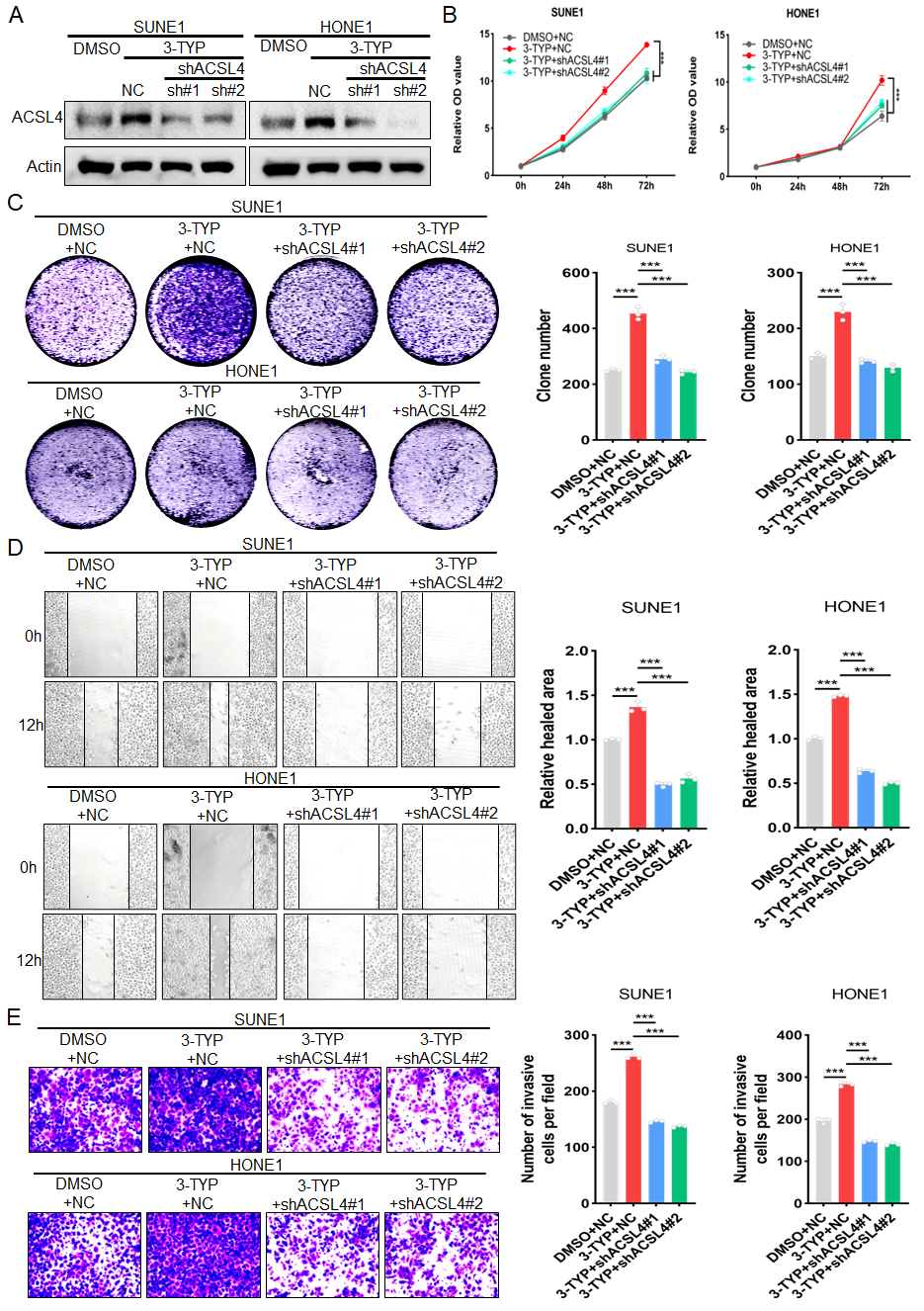


**Supplementary Fig.S11 SIRT3 inhibits ACSL4-mediated malignant progression of NPC cells.**

SUNE1-NC, SUNE1-shACSL4#1, SUNE1-shACSL4#2 and HONE1-NC, HONE1-shACSL4#1, HONE1-shACSL4#2 cells treated with 3-TYP (50μM), (A) Western blot was used to detect ACSL4 protein expression, (B) CCK8 and (C) colony formation assay were used to analyze cell proliferation. (D) Scratch assay was used to detect the cell migration. (E) Transwell assay was used to detect the cell invasion. The error line is expressed as mean ± SD. ***p<0.001.


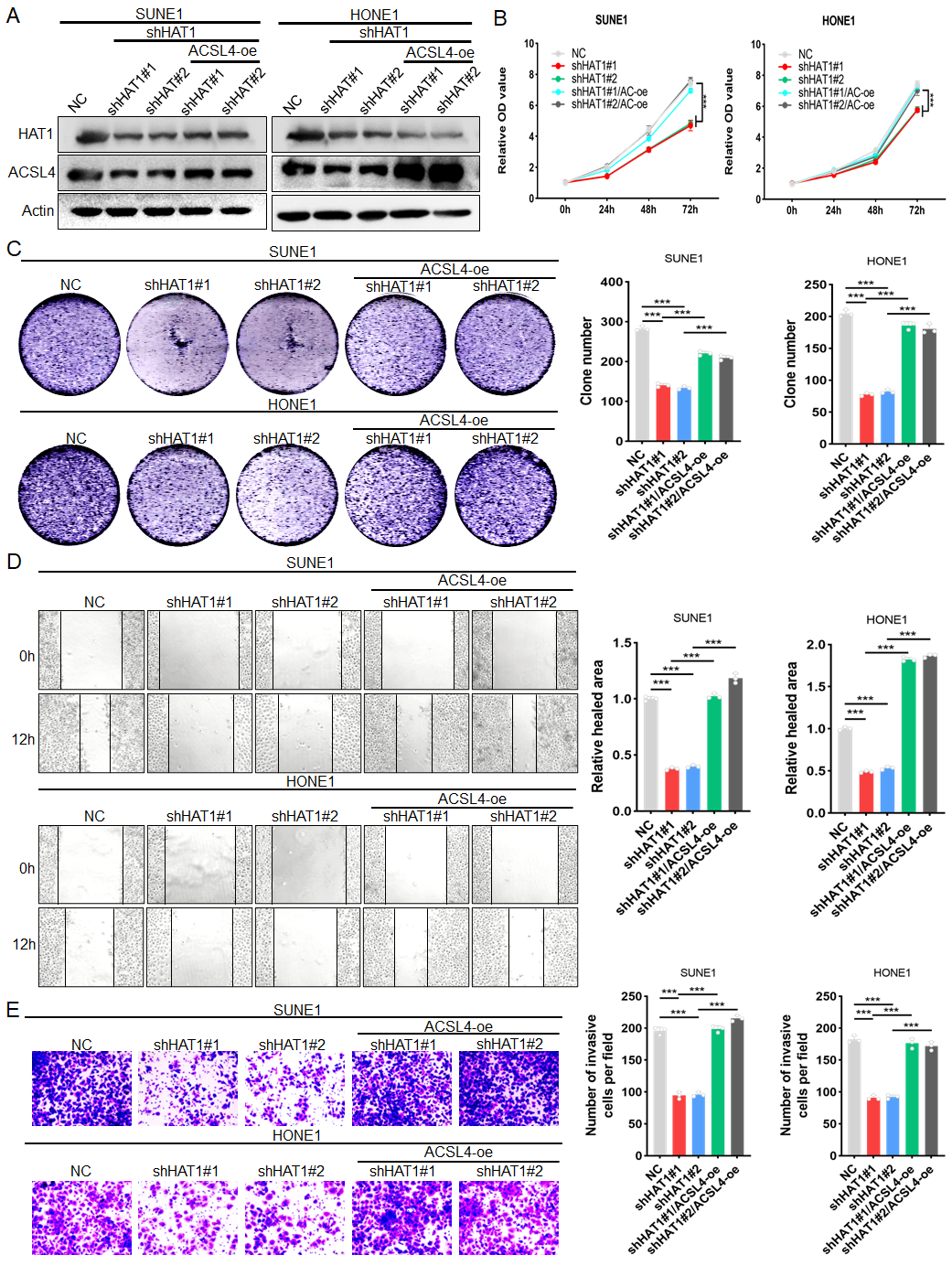


**Supplementary Fig.S12 HAT1 promotes ACSL4-mediated malignant progression of NPC cells.**

SUNE1-NC, SUNE1-shHAT1#1, SUNE1-shHAT1#2 and HONE1-NC, HONE1-shHAT1#1, HONE1-shHAT1#2 cells were transfected with pcDNA-Flag-ASCL4 plasmid. (A) Western blot was used to detect the protein expression of HAT1 and ACSL4. (B) CCK8 and (C) colony formation assay were used to analyze cell proliferation. (D) Scratch assay was used to detect the cell migration. (E) Transwell assay was used to detect the cell invasion. Negative control (NC). The error line is expressed as mean ± SD. ***p<0.001.


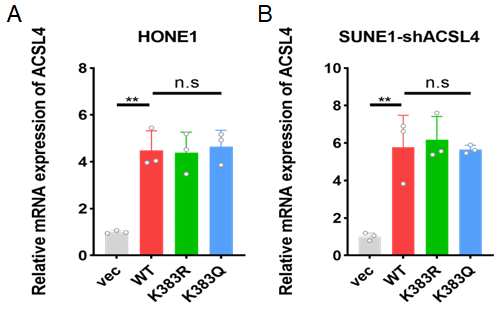


**Supplementary Fig.S13 The K383 mutation of ACSL4 does not affect its mRNA level.** Using the SUNE1-shACSL4-vec, SUNE1-shACSL4-WT, SUNE1-shACSL4-K383R, SUNE1-shACSL4-K383Q cells, and HONE1-vec, HONE1-WT, HONE1-K383R, HONE1-K383Q cells, qPCR assay was used to detect ACSL4 mRNA. Blank plasmid (vec). The error line is expressed as mean ± SD. **p<0.01.


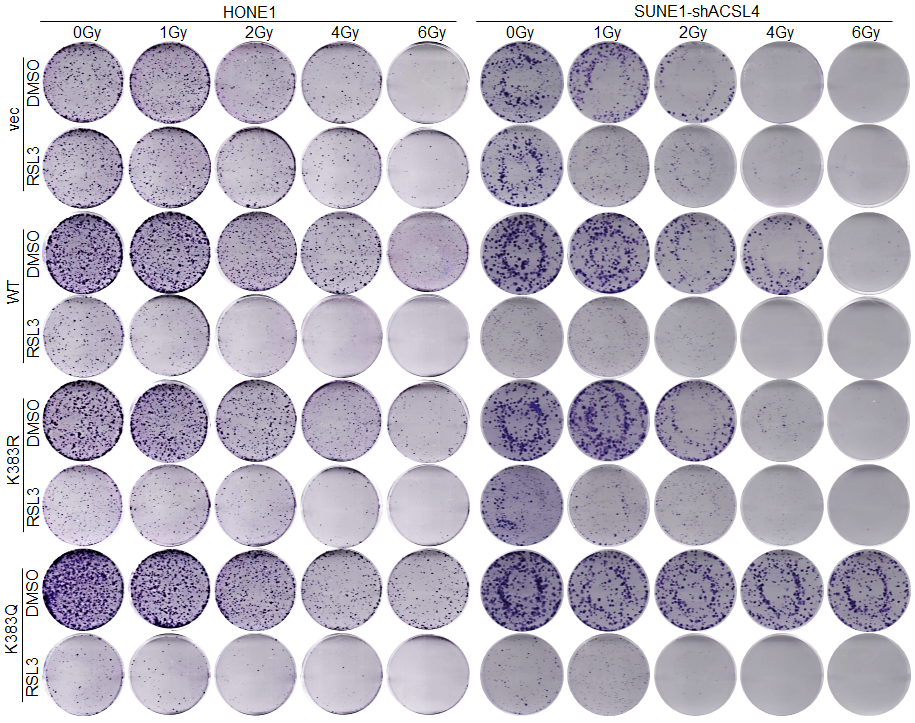


**Supplementary Figure S14. K383 acetylation of ACSL4 enhances radiosensitivity of NPC cells by inducing ferroptosis.** SUNE1-shACSL4-vec, SUNE1-shACSL4-WT, SUNE1-shACSL4-K383R, SUNE1-shACSL4-K383Q cells and HONE1-vec, HONE1-WT, HONE1-K383R, HONE1-K383Q cells (3×10^3^ cells/well) were used. The radioresistance of NPC cells was detected by colony formation assay under different doses (0, 1, 2, 4, 6Gy) of IR and treatment with RSL3 (5μM).


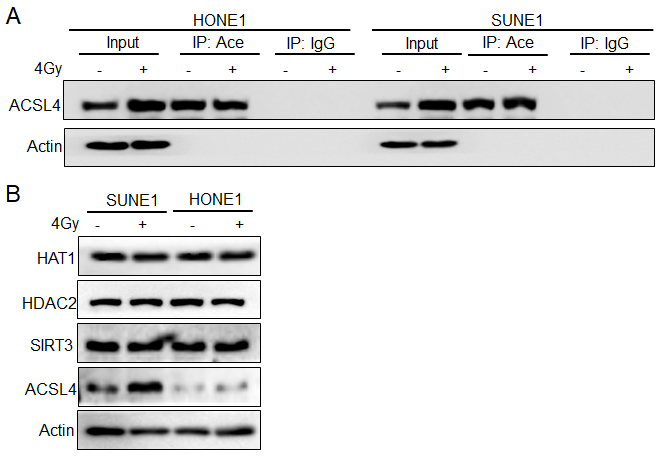


**Supplementary Figure S15.** **IR can increase the protein expression of ACSL4 without affecting its acetylation.** NPC cells were treated with 4 Gy of IR. (A) Western blotting and IP experiments were performed to assess the protein expression and acetylation of ACSL4. (B) Western blotting was used to evaluate the protein expression of HAT1, HDAC2, SIRT3, and ACSL4.

**Supplementary Tables**

**Table.S1 Primers for homologous recombination**

| ID | Primer (5’-3’) |
| --- | --- |
| pcDNA-Flag-ASCL4-F | tagtccagtgtggtggaattcATGAAACTTAAGCTAAATGTGCTCACC |
| pcDNA-Flag-ASCL4-R | atggtgatgatggaagggcccTTTGCCCCCATACATTCGTT |
| pcDNA-His-SIRT3-F | tagtccagtgtggtggaattcATGGCGTTCTGGGGTTGG |
| pcDNA-His-SIRT3-R | atggtgatgatggaagggcccTTTGTCTGGTCCATCAAGCTTCC |
| pcDNA-His-HAT1-F | tagtccagtgtggtggaattcATGGCGGGATTTGGTGCT |
| pcDNA-His-HAT1-R | atggtgatgatggaagggcccCTCTTGAGCAAGTCGTTCAATAACA |
| pcDNA-His-FBXO10-F | tagtccagtgtggtggaattcATGGAGGCTGGTGGCCTC |
| pcDNA-His-FBXO10-R | atggtgatgatggaagggcccCAGGATGGTGCAGAAGACACTG |
| PCDH-Flag-ASCL4-F | gattctagagctagcgaattcATGAAACTTAAGCTAAATGTGCTCACC |
| PCDH-Flag-ASCL4-R | cggaaggcgaacatggggcccTTTGCCCCCATACATTCGTT |
| ASCL4-K383R-F | atagagaatgttatgagcaaagtGAATGTTATGAGCAAAGTCCAAGAGA |
| ASCL4-K383R-R | gctcataacattctctataaattctatccatgattTATAAATTCTATCCATGATTTCCGGA |
| ASCL4-K383Q-F | tatcagaatgttatgagcaAGAATGTTATGAGCAAAGTCCAAGAG |
| ASCL4-K383Q-R | gctcataacattctgataaattctatccatgATAAATTCTATCCATGATTTCCGGA |
| SIRT3-H248Y-F | CTGGTTGAAGCTTATGGAACCTTTGCCTCTGCC |
| SIRT3-H248Y-R | CCATAAGCTTCAACCAGCTTTGAGGCAGGGAT |

**Table.S2 Target sequences for RNA interference**

| ID | Target sequence (5’ - 3’) |
| --- | --- |
| shACSL4#1 | GCAGCTTCCAATGCCTCTTGT |
| shACSL4#2 | GCAGTAGTTCATGGGCTAAAT |
| shHAT1#1 | GCAAGGATTCAATGAAGATAT |
| shHAT1#2 | CCGTGTTGAATATGCATCTAA |
| shHDAC1 | CGTTCTTAACTTTGAACCATA |
| shHDAC2 | GACGGTATCATTCCATAAATA |
| shHDAC3 | CCTTCCACAAATACGGAAATT |
| shSIRT3 | GTGGGTGCTTCAAGTGTTGTT |
| NC | TTCTCCGAACGTGTCACGT |

**Table.S3 Primers for qPCR**

| ID | Target sequence (5’ - 3’) |
| --- | --- |
| ACSL4-F | ACCAGGGAAATCCTAAGTGAAG |
| ACSL4-R | GGTGTTCTTTGGTTTTAGTCCC |
| HDAC2-F | CGAGCATCAGACAAGCGGATAGC |
| HDAC2-R | AGCCACATTTCTTCGACCTCCTTC |
| SIRT1-F | TCTTGTGGCAGTAACAGTGATAGTG |
| SIRT1-R | TGGAACATCAGGCTCATCTTCTAAG |
| SIRT2-F | TTCAAGCCAACCATCTGTCACTAC |
| SIRT2-R | CCTCCACCAAGTCCTCCTGTTC |
| SIRT3-F | GCCATTTTTGAACTCCCATTCT |
| SIRT3-R | GGAGAAAGTAGTGAGTGACGTT |
| SIRT4-F | CATCCAGCATGGTGATTTTGTC |
| SIRT4-R | AGTACAGCTTTCCGAGTTTCTC |
| SIRT5-F | AGATTGTCCCAAGTCGATTGAT |
| SIRT5-R | CCATTTTCAGGCAAATCTGGTT |
| SIRT6-F | CTCCTCCGCTTCCTGGTCAG |
| SIRT6-R | CGTCTTACACTTGGCACATTCTTC |
| SIRT7-F | TGCCCTCCACAGACACCAGAC |
| SIRT7-R  ACTIN-F | CGCCGCTTCCCAGTTCAAAGG CATGTACGTTGCTATCCAGGC |
| ACTIN-R | CTCCTTAATGTCACGCACGAT |

**Table S4.** **Clinical characteristics of NPC patients (pathological sections)**

| Characteristics | Number of patients (%) |
| --- | --- |
| **Gender** |  |
| Male | 28（70.00%） |
| Female | 12（30.00%） |
| **Age** |  |
| ≥45 | 34（85.00%） |
| ＜45 | 6（15.00%） |
| **Pathological type** |  |
| Undifferentiated | 35（87.50%） |
| Differentiated | 5（12.50%） |
