# Supplementary material for: HAT1/HDAC2 mediated ACSL4 acetylation confers radiosensitivity by inducing ferroptosis in nasopharyngeal carcinoma
Source: Cell Death Dis. 2025 Mar 6;16(1):160. doi: 10.1038/s41419-025-07477-4 (PMC11885570; doi:10.1038/s41419-025-07477-4)

**Original Data**

**HAT1/HDAC2 mediated ACSL4 acetylation confers radiosensitivity by inducing ferroptosis in nasopharyngeal carcinoma**

Peijun Zhou, Xingzhi Peng, Kun zhang, Jin Cheng, Min Tang, Lin Shen, Qin Zhou, Dan Li, Lifang Yang

**qPCR data**


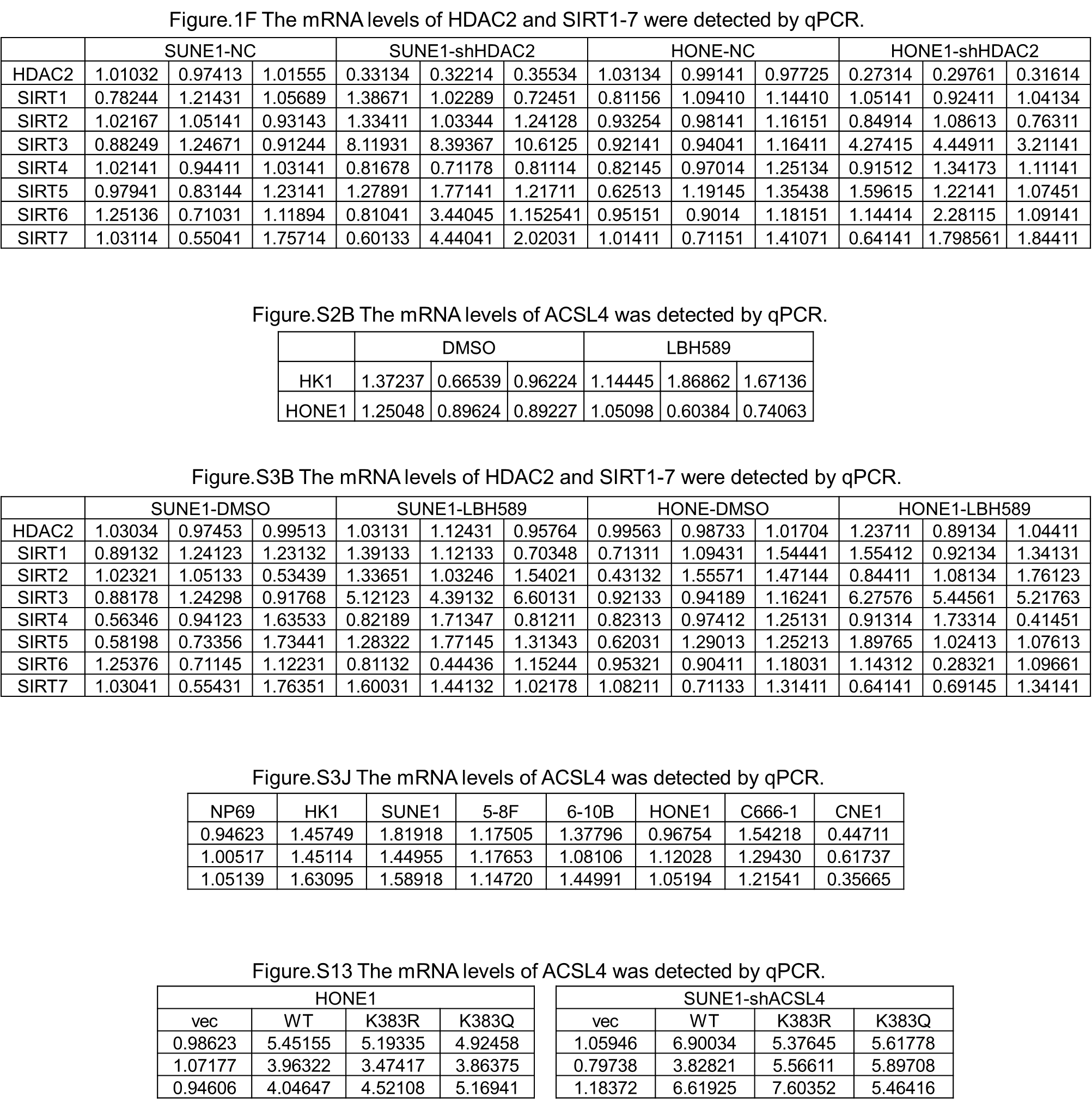


**uncropped western blots**


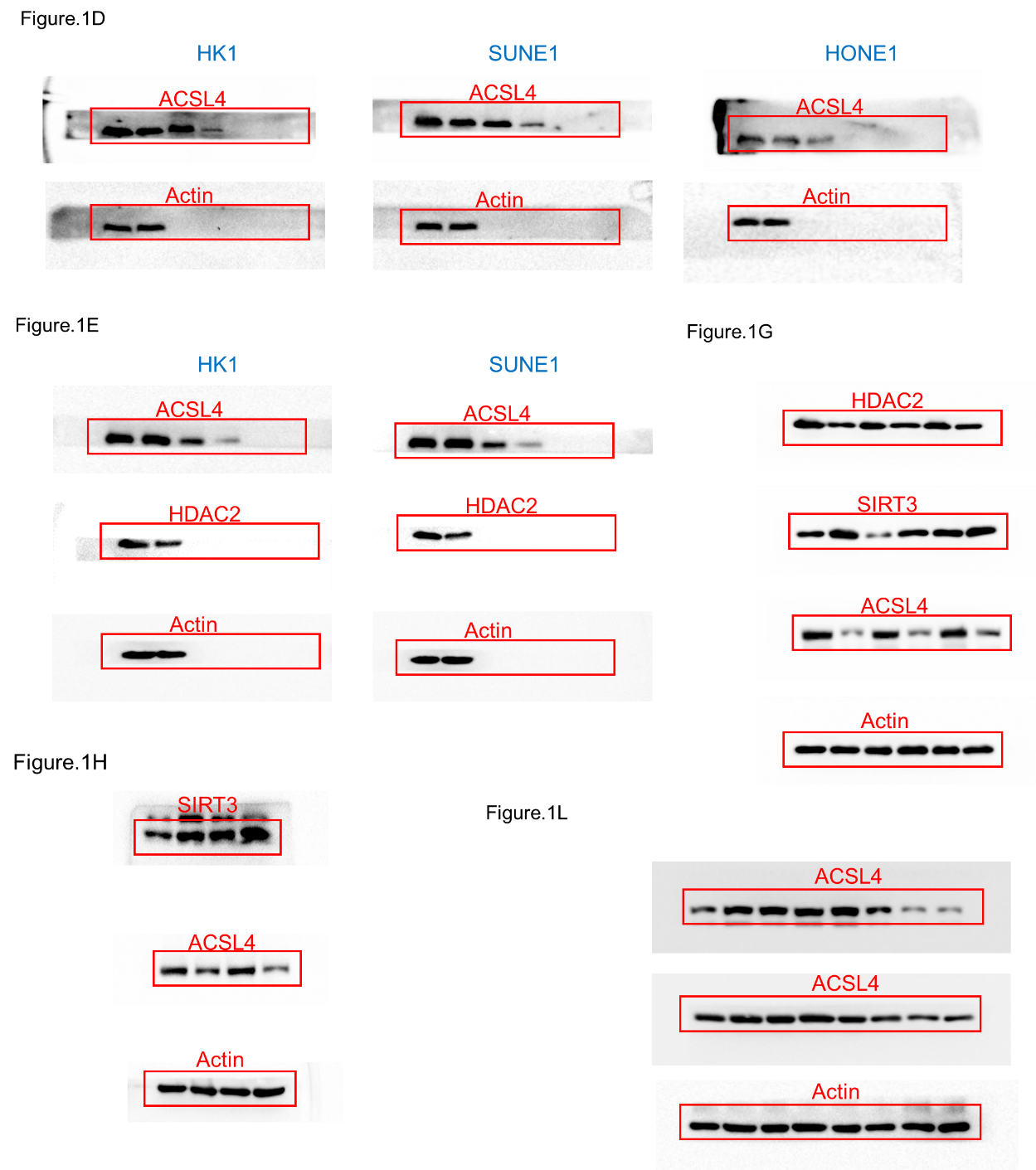


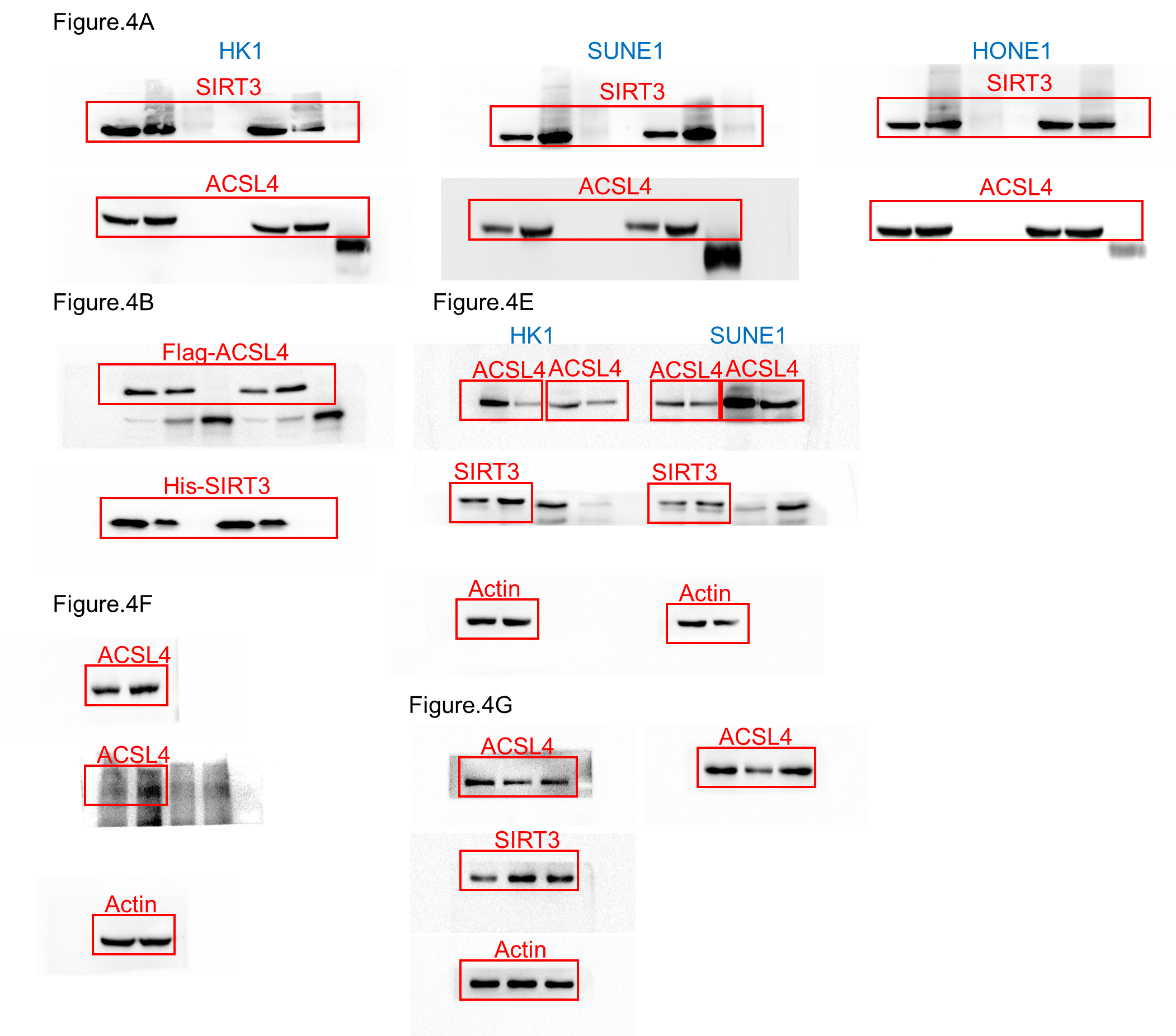

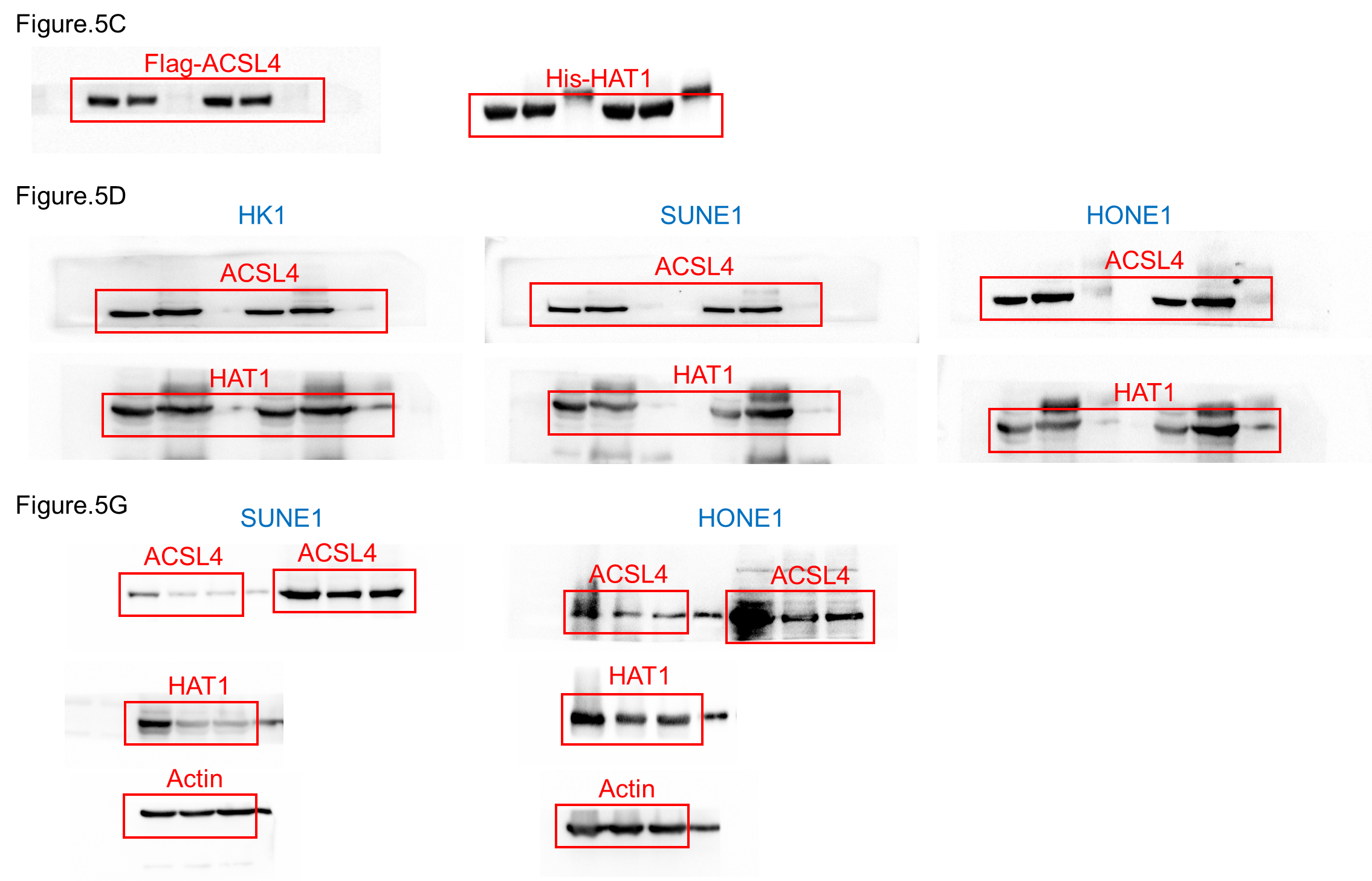

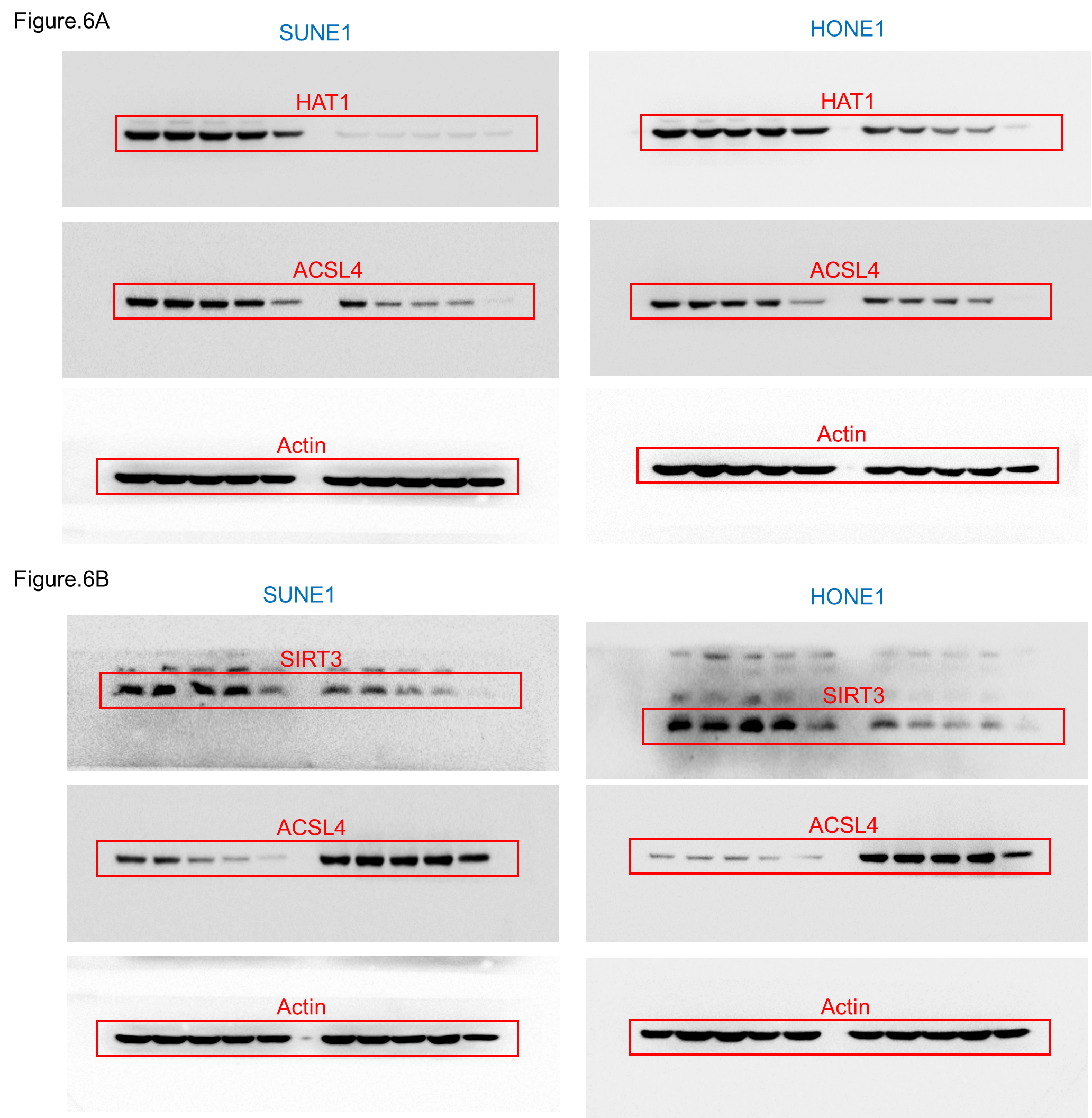

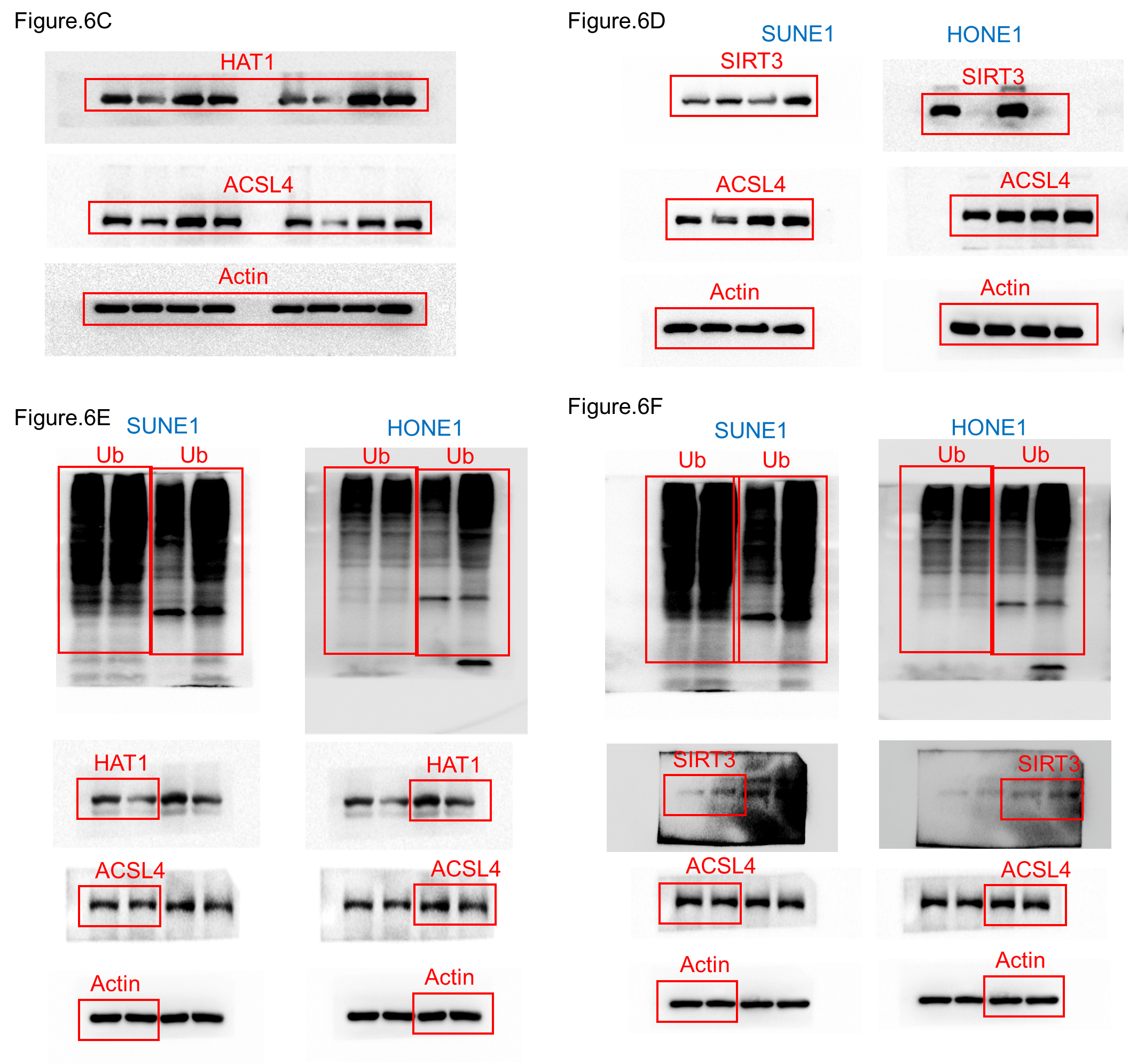

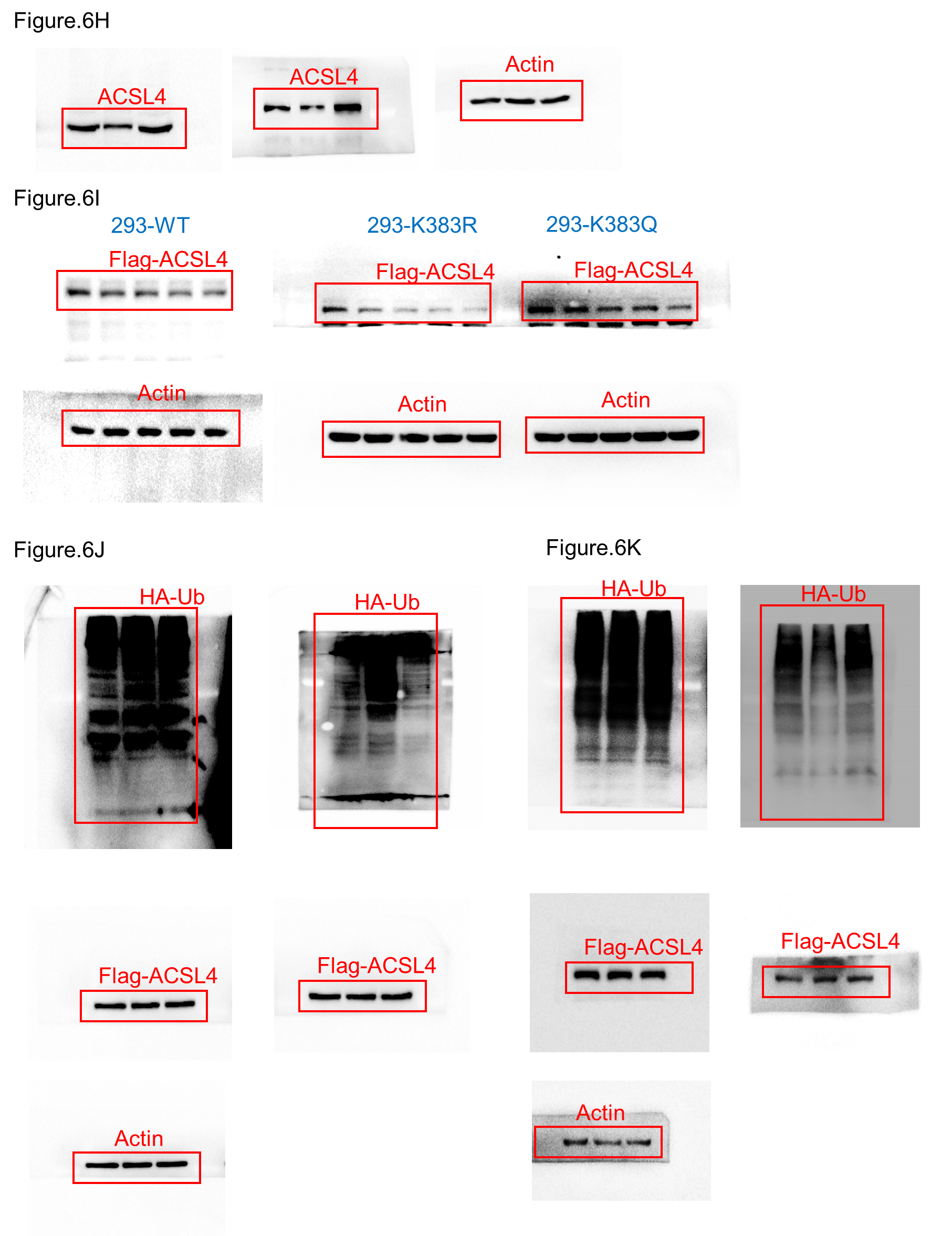

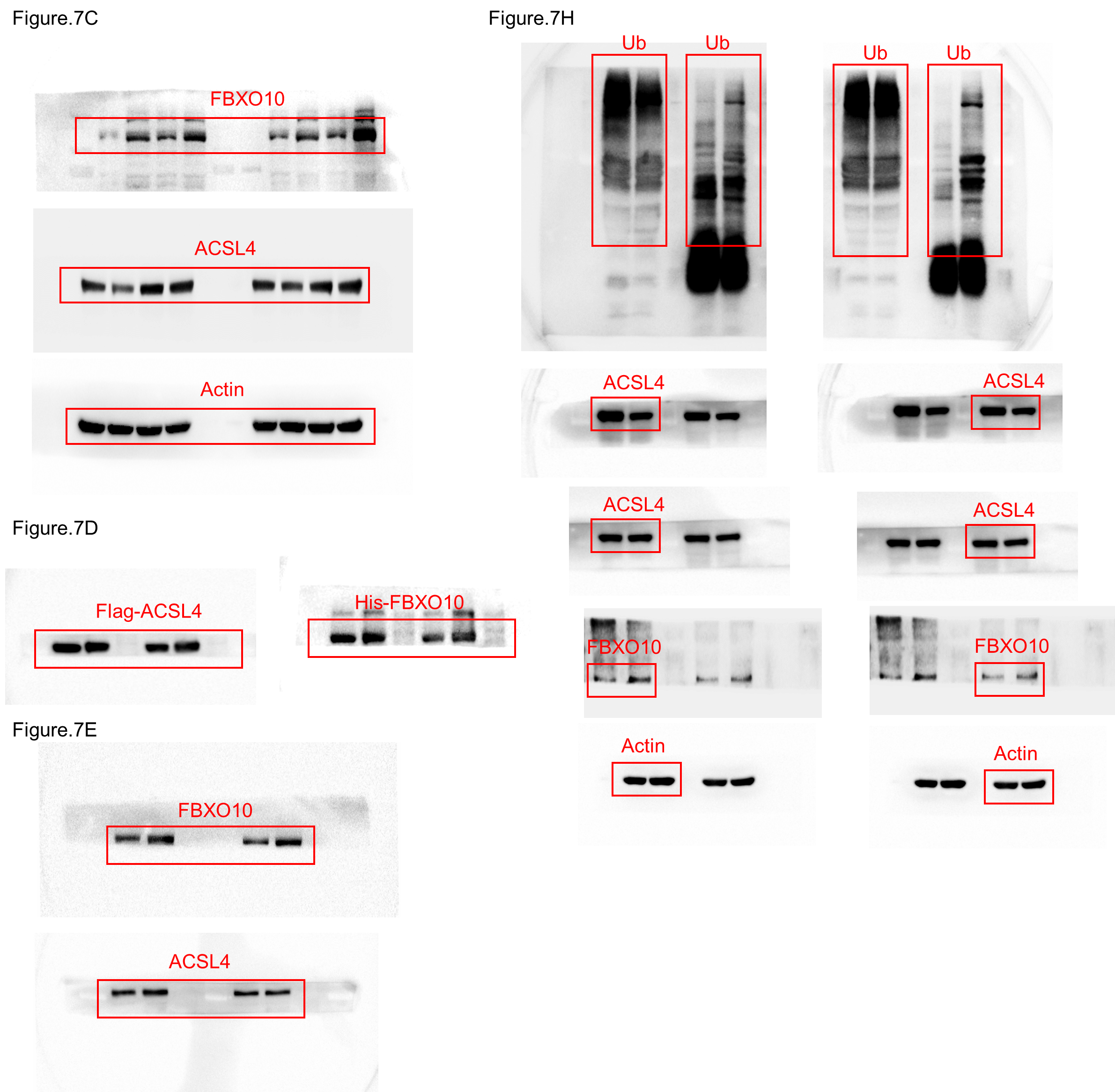

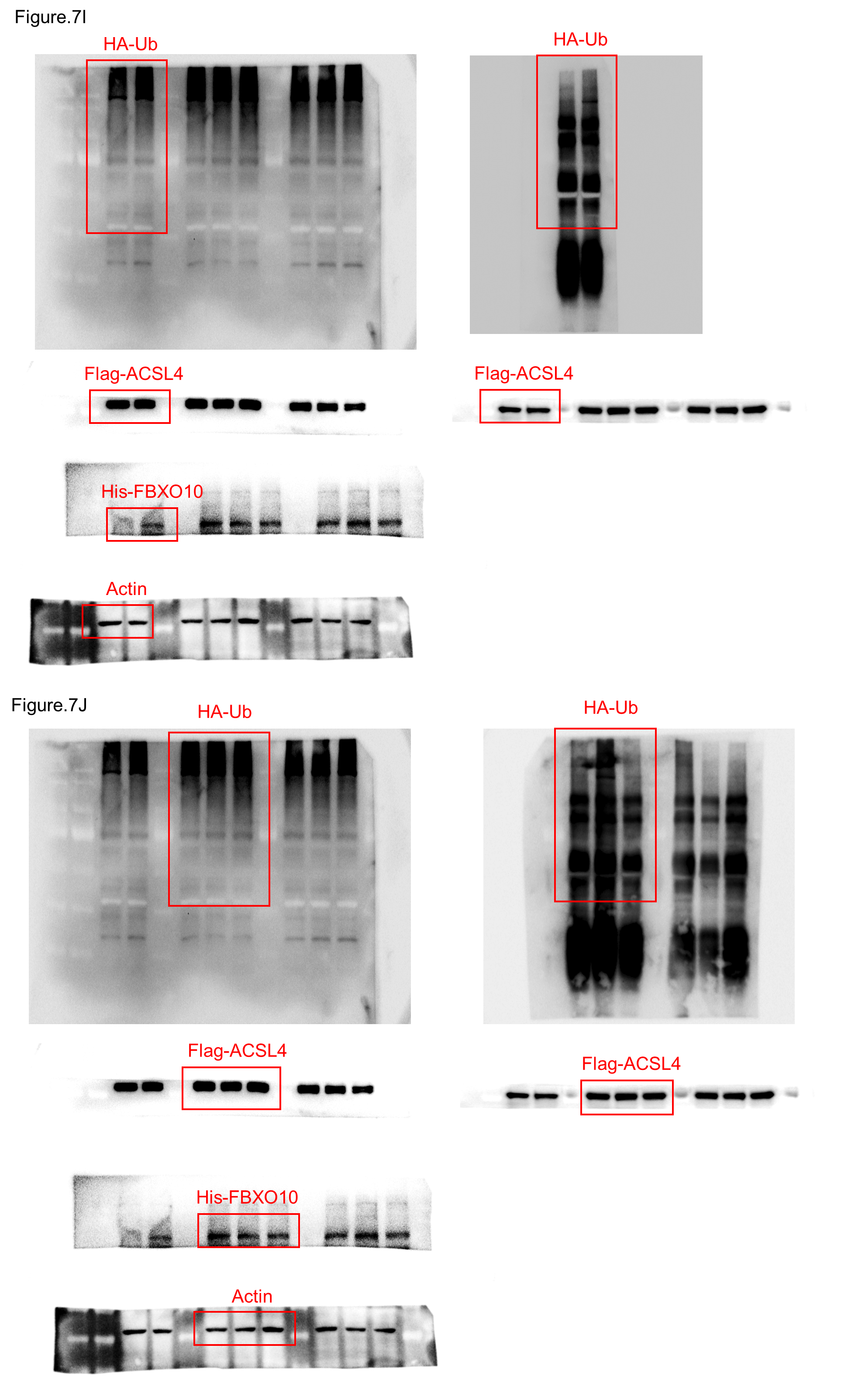

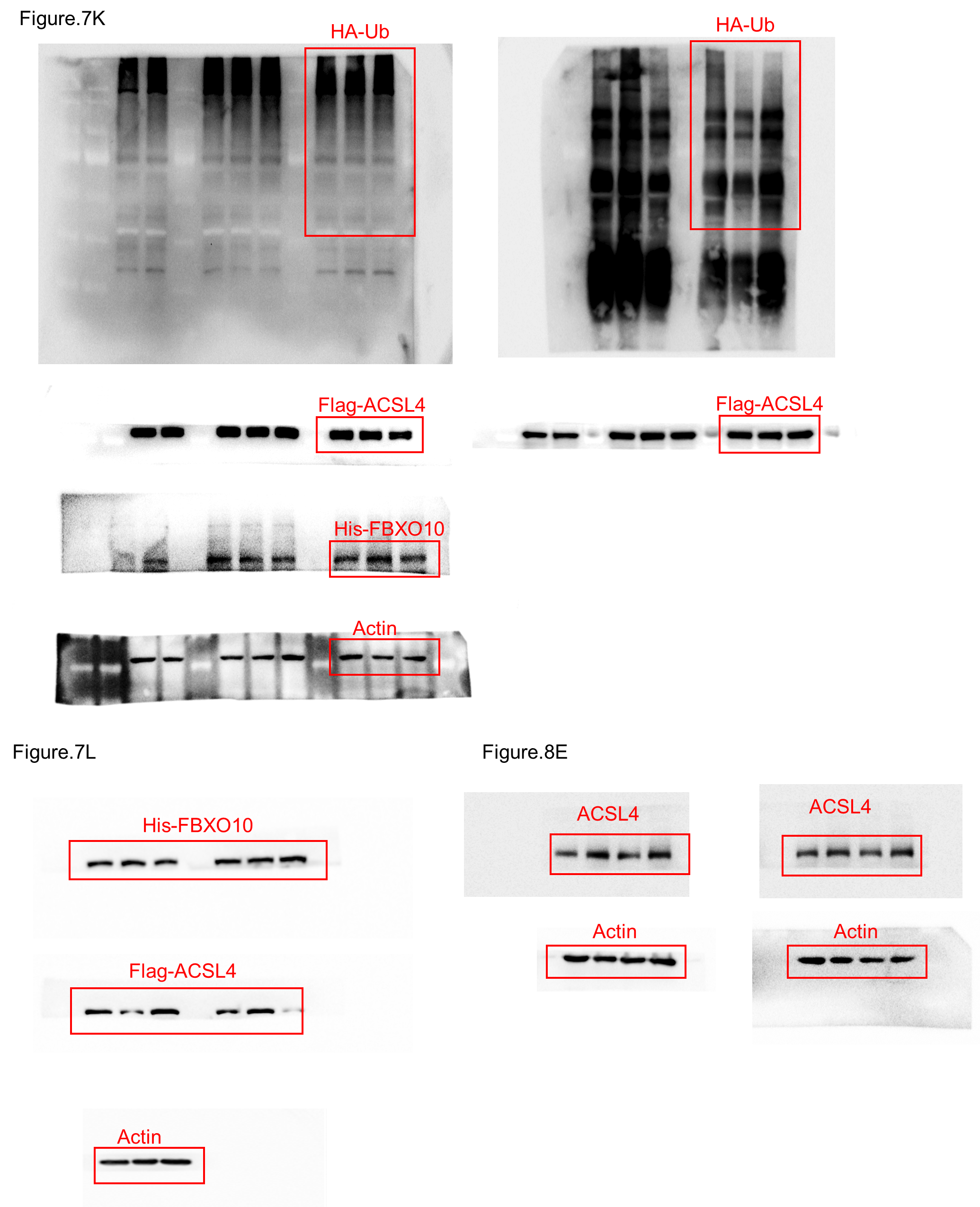

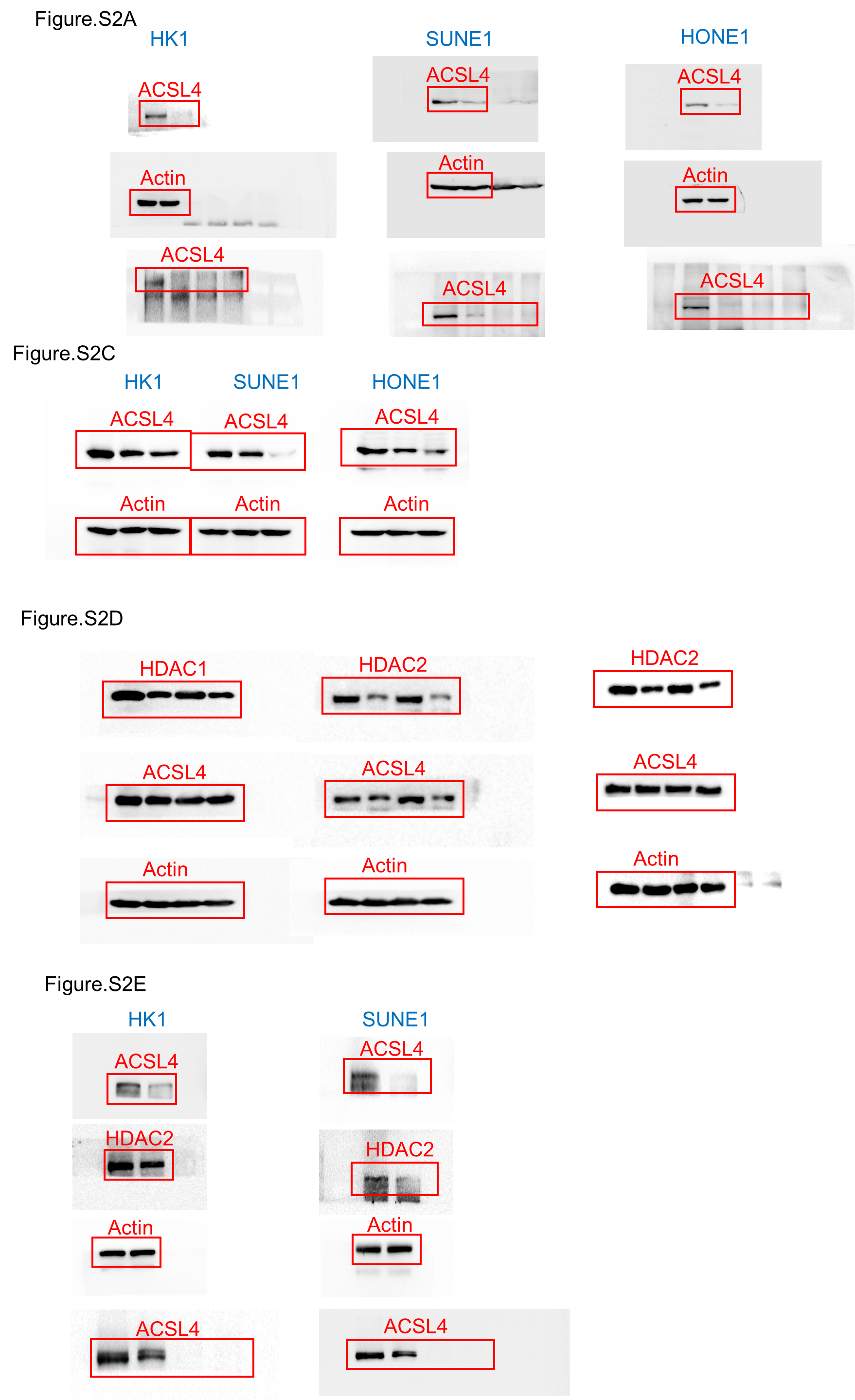


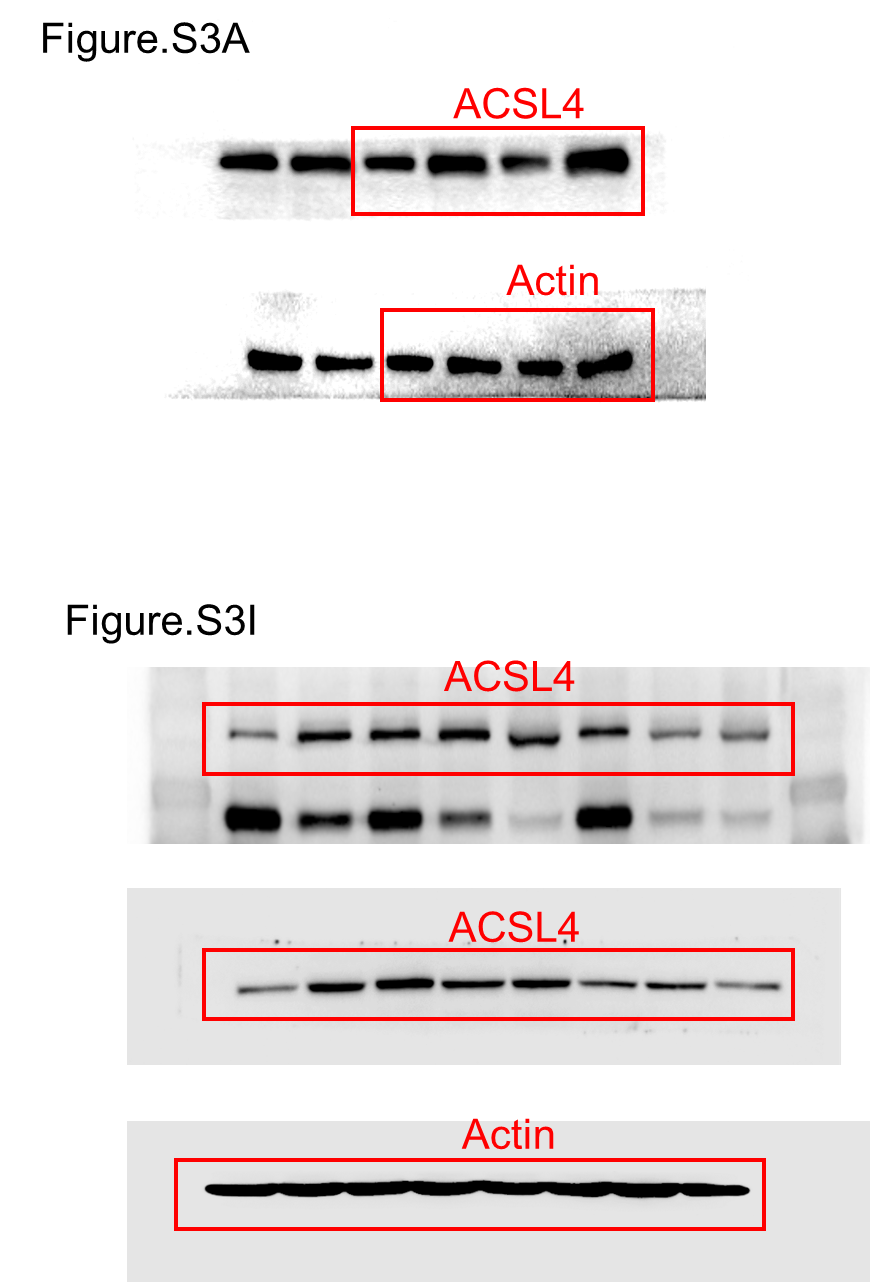


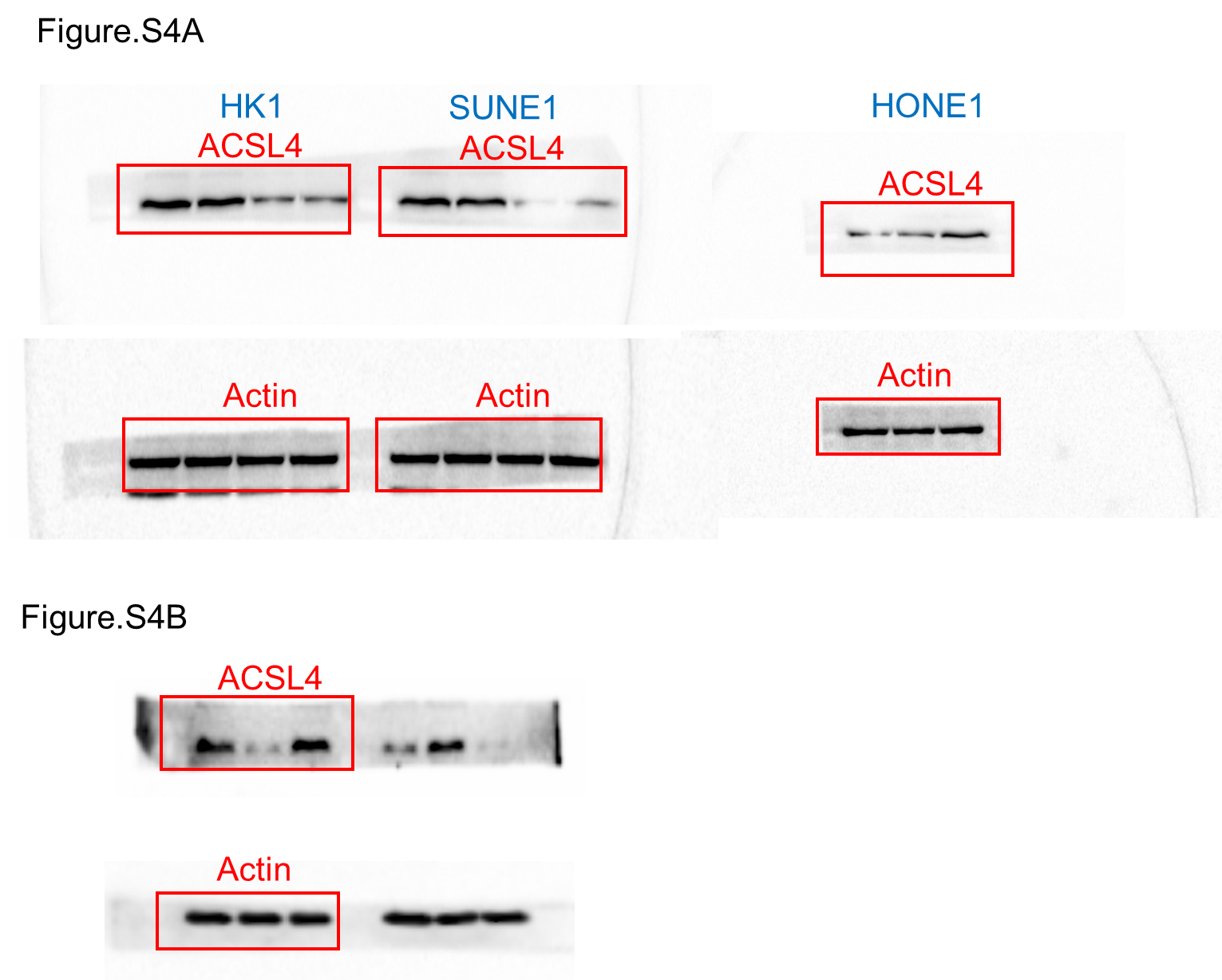

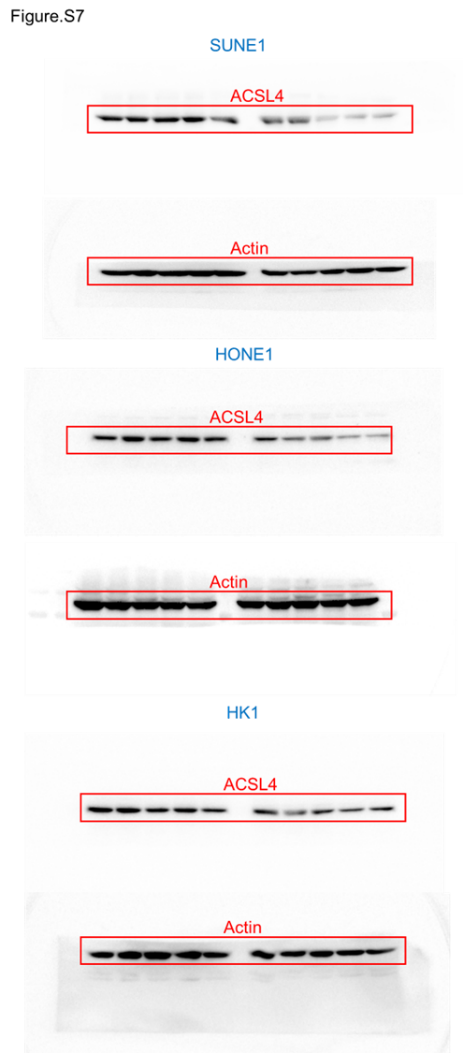

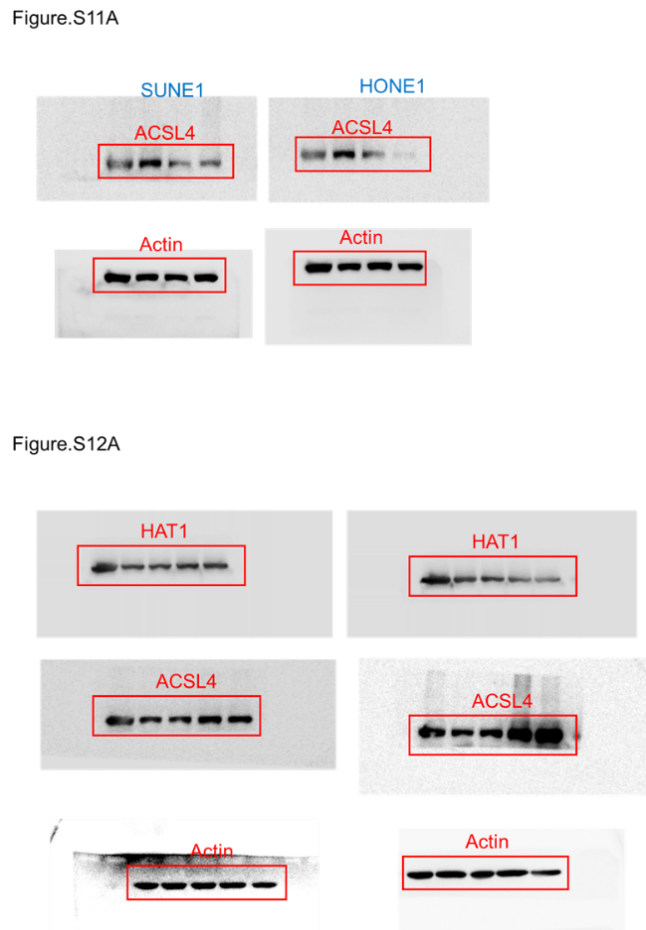

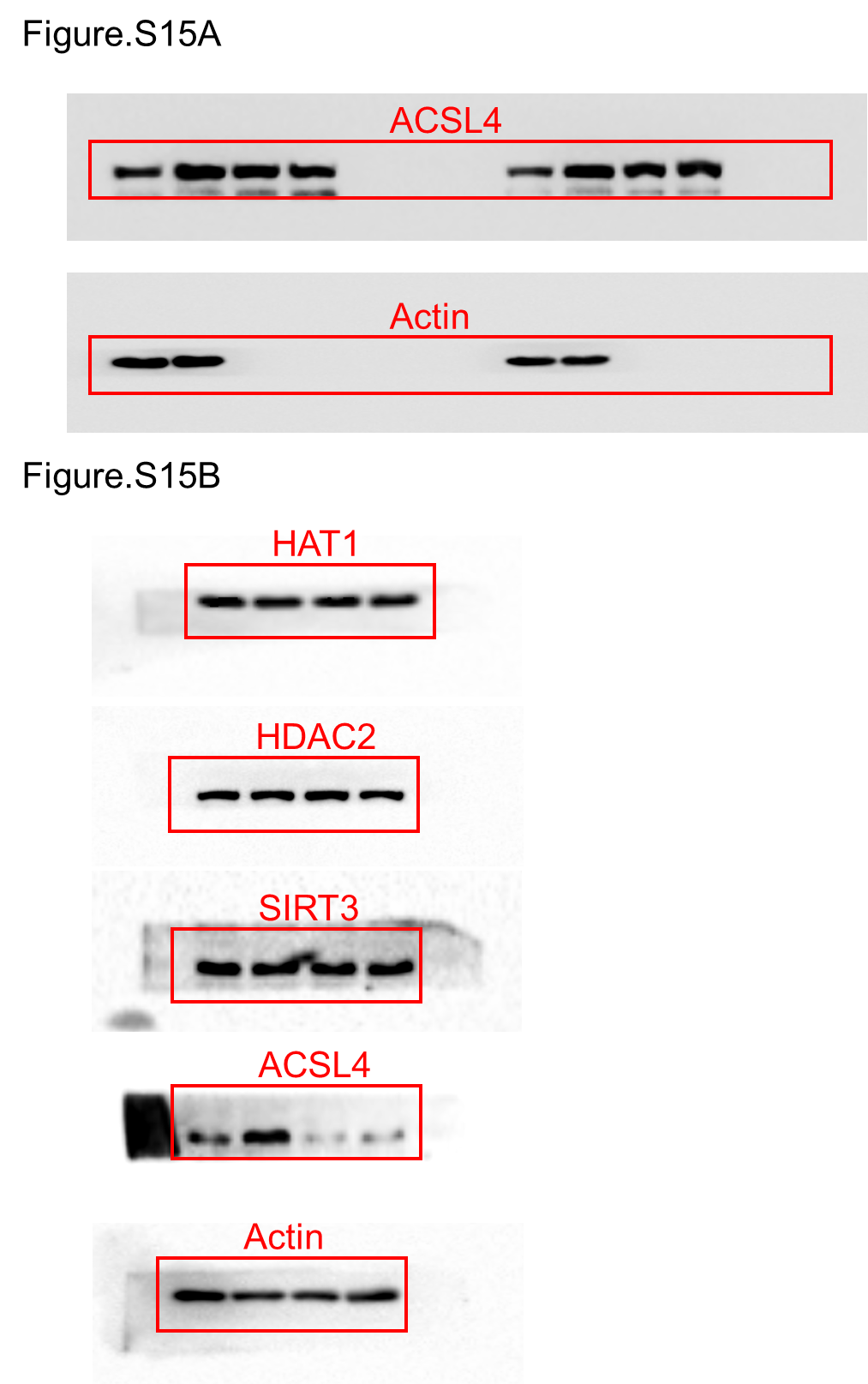

Supplement: Supplementary file 2 — Original Data [file 41419_2025_7477_MOESM2_ESM.docx]
